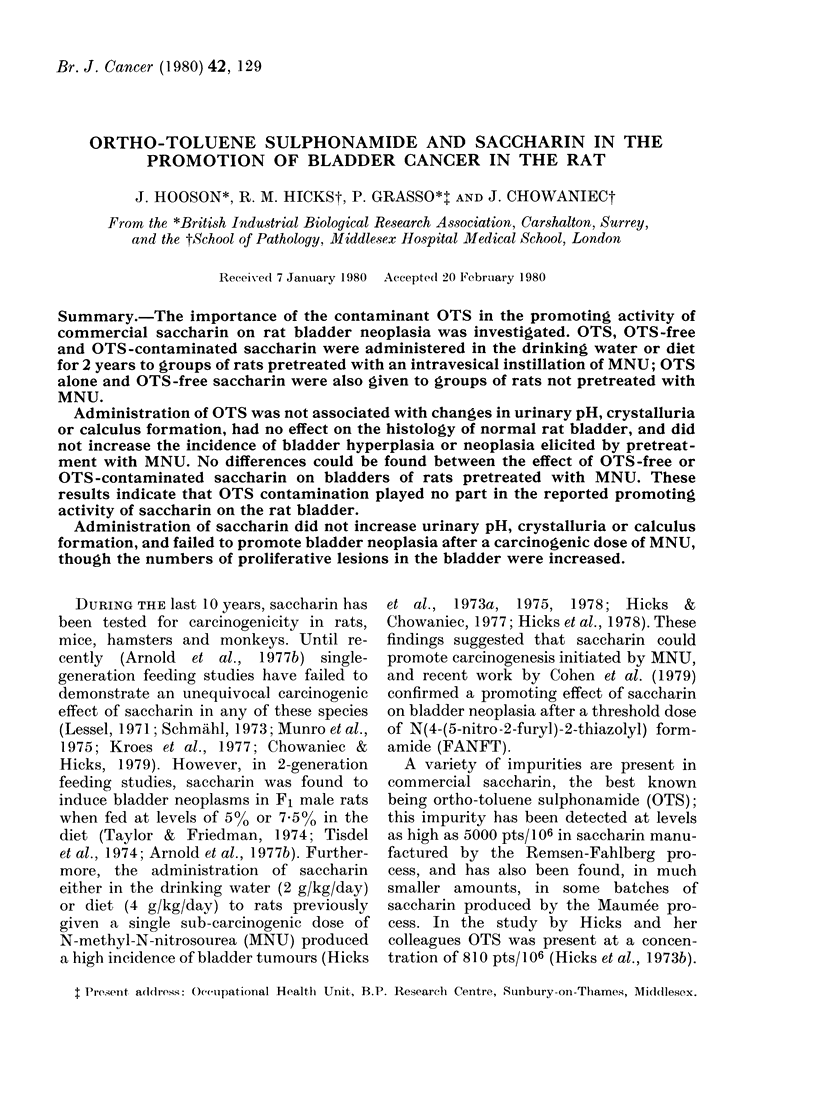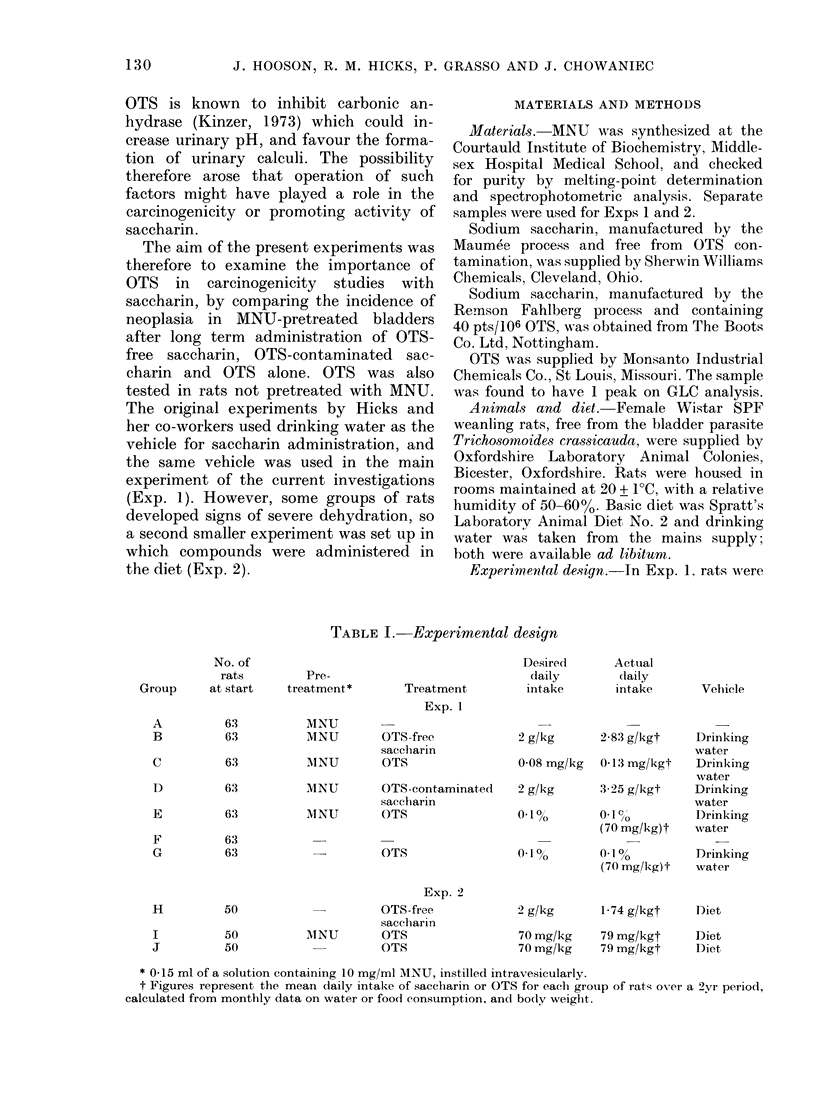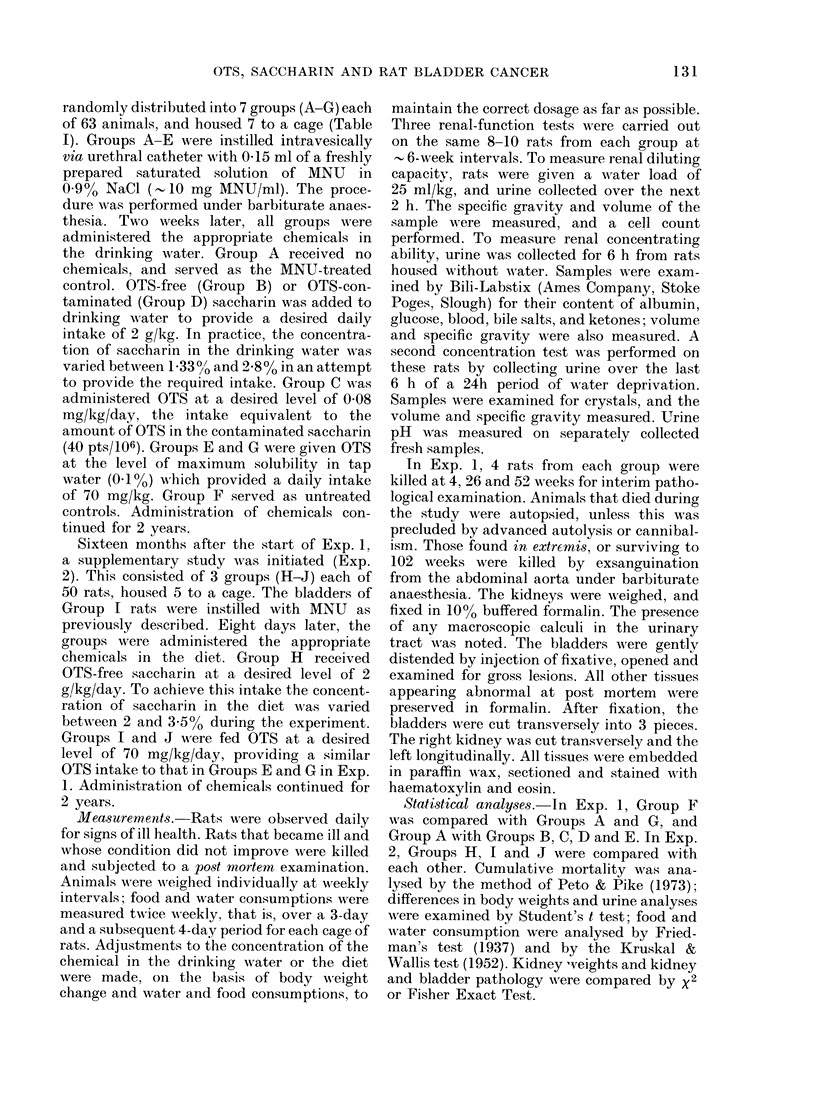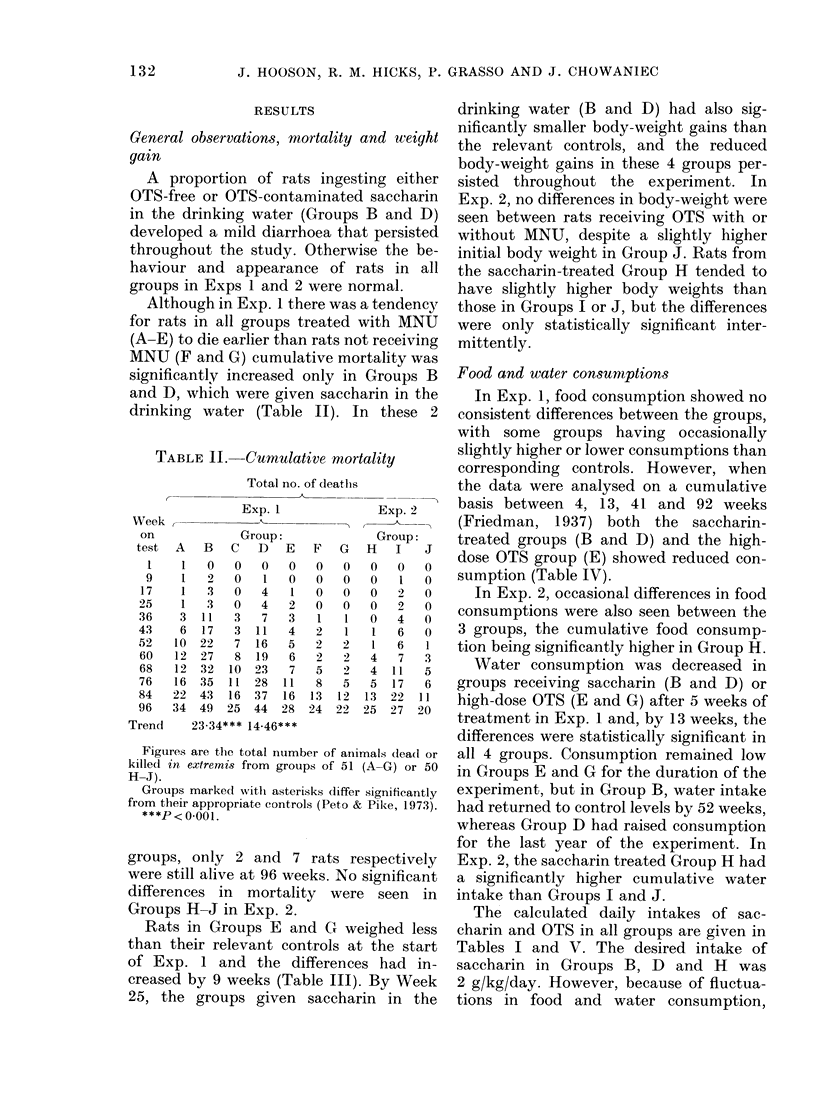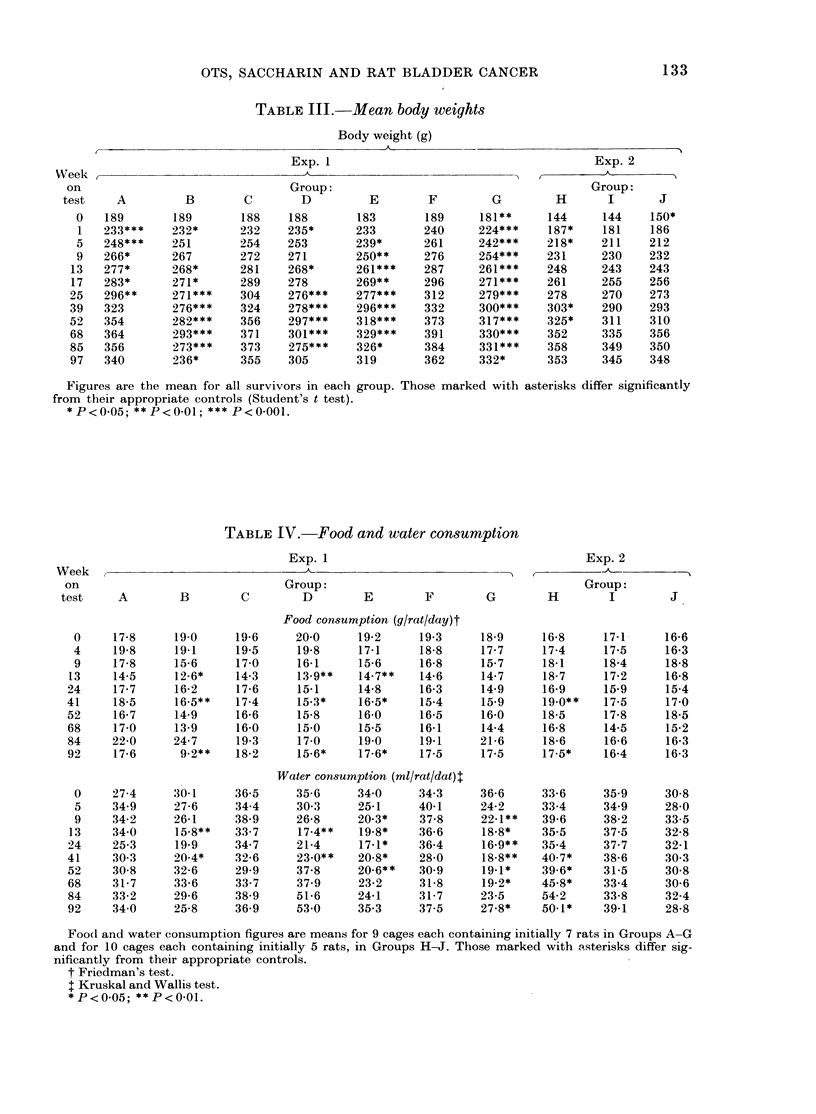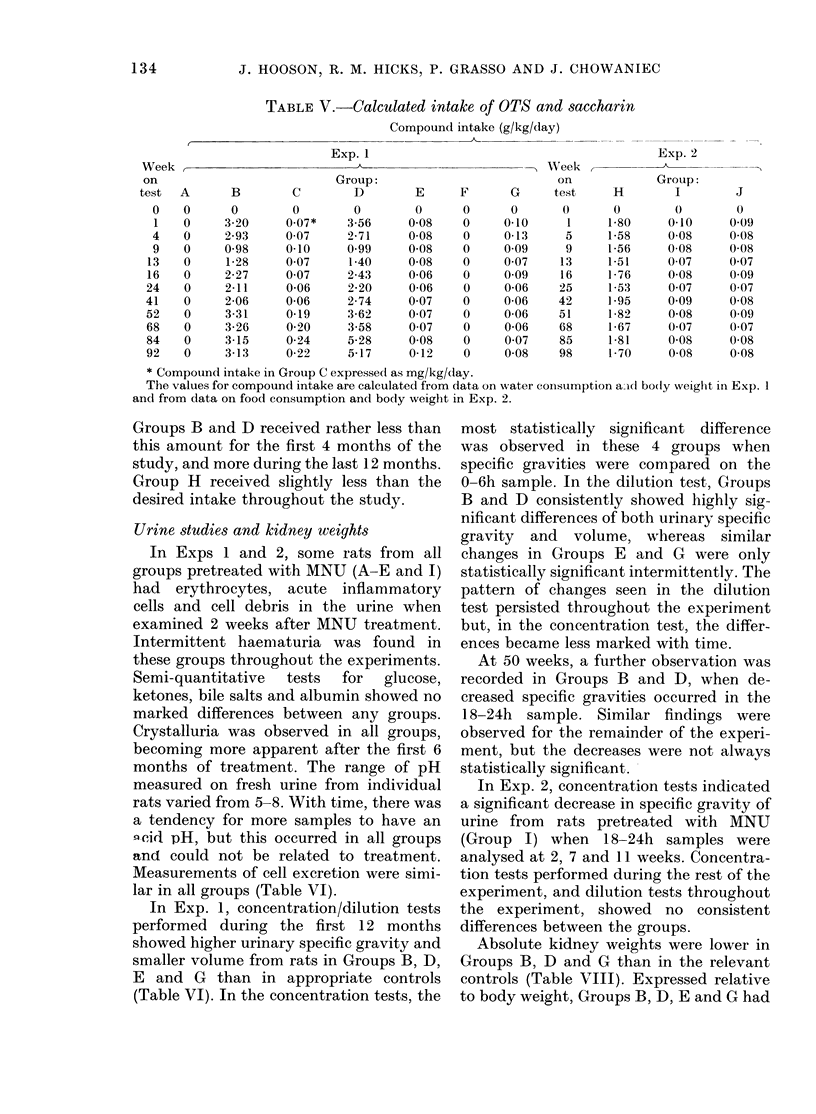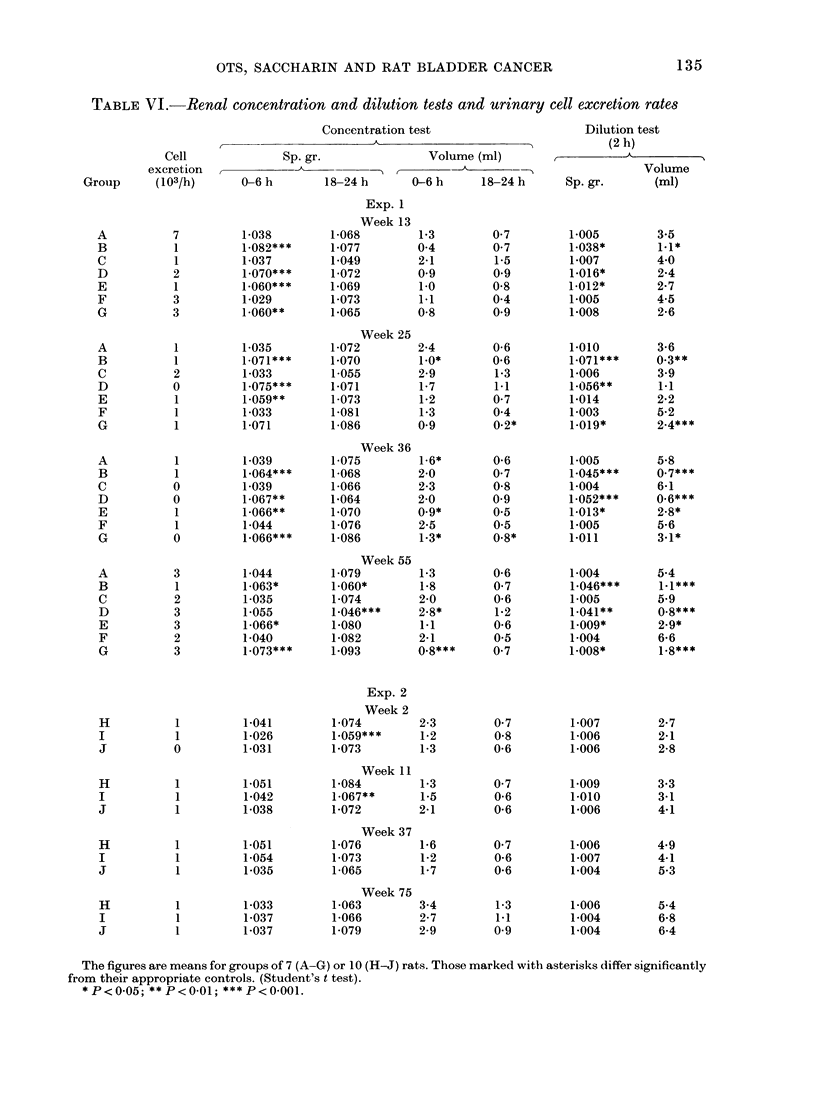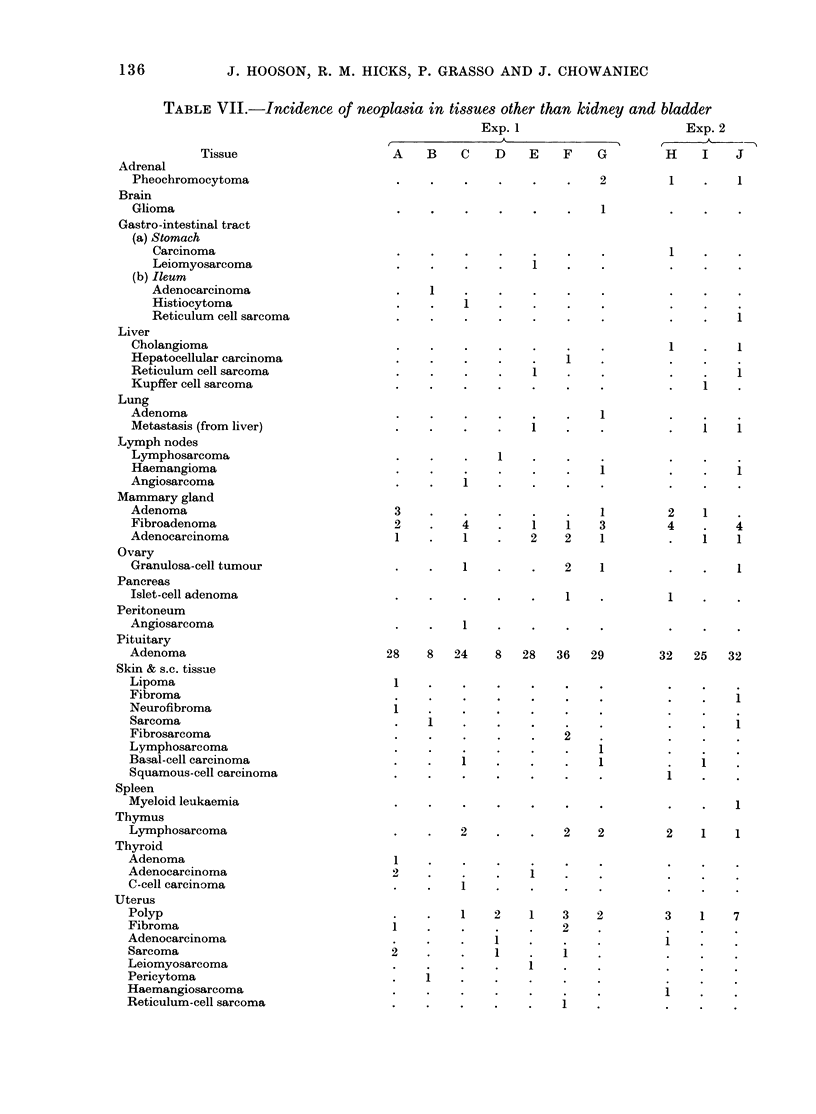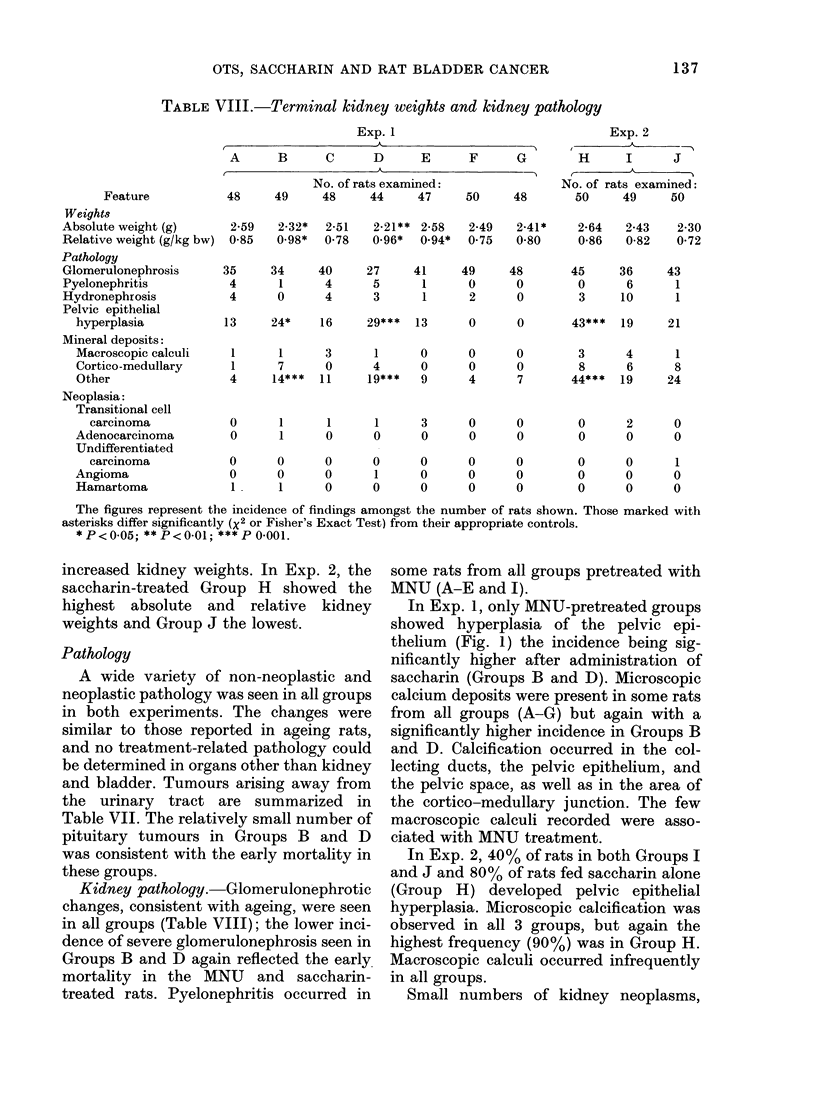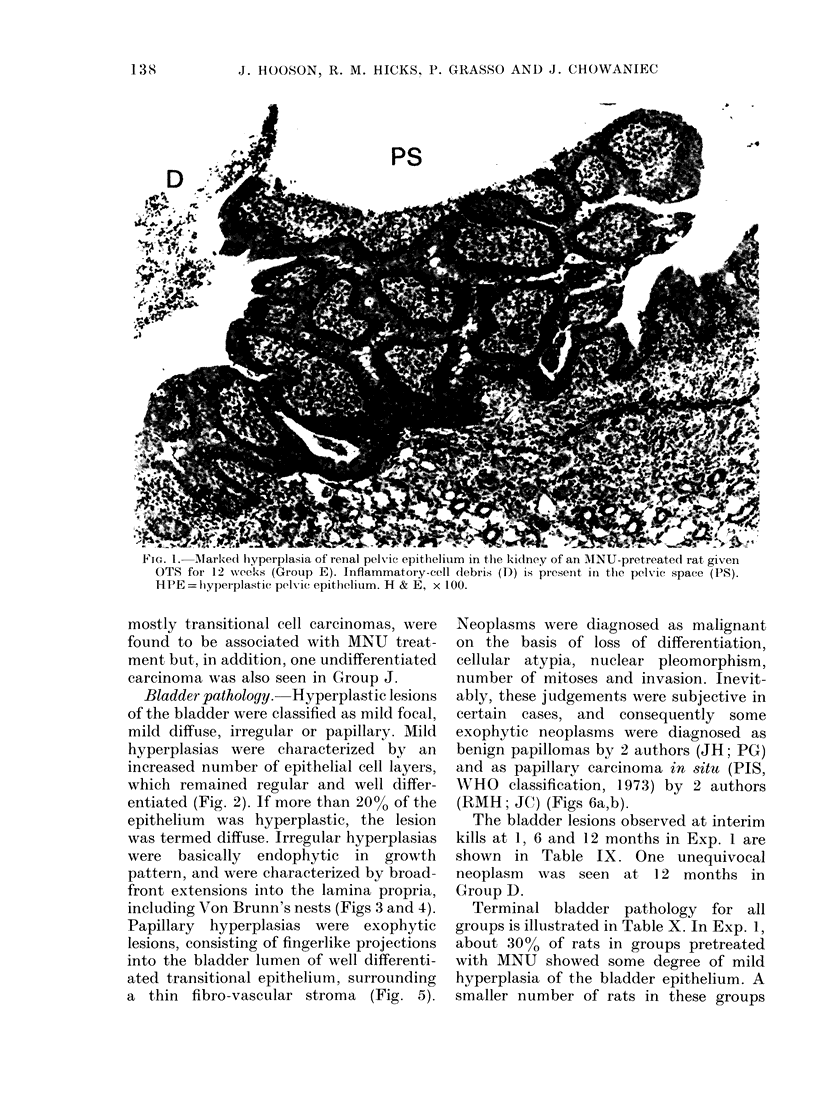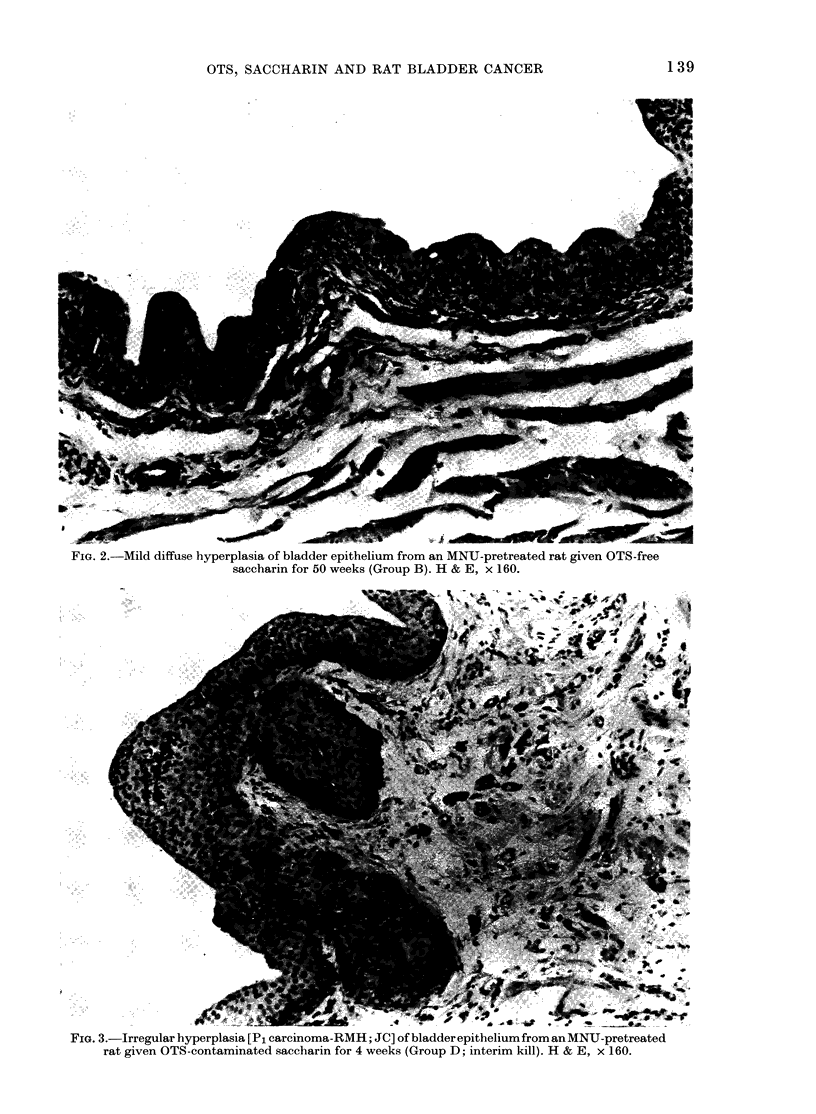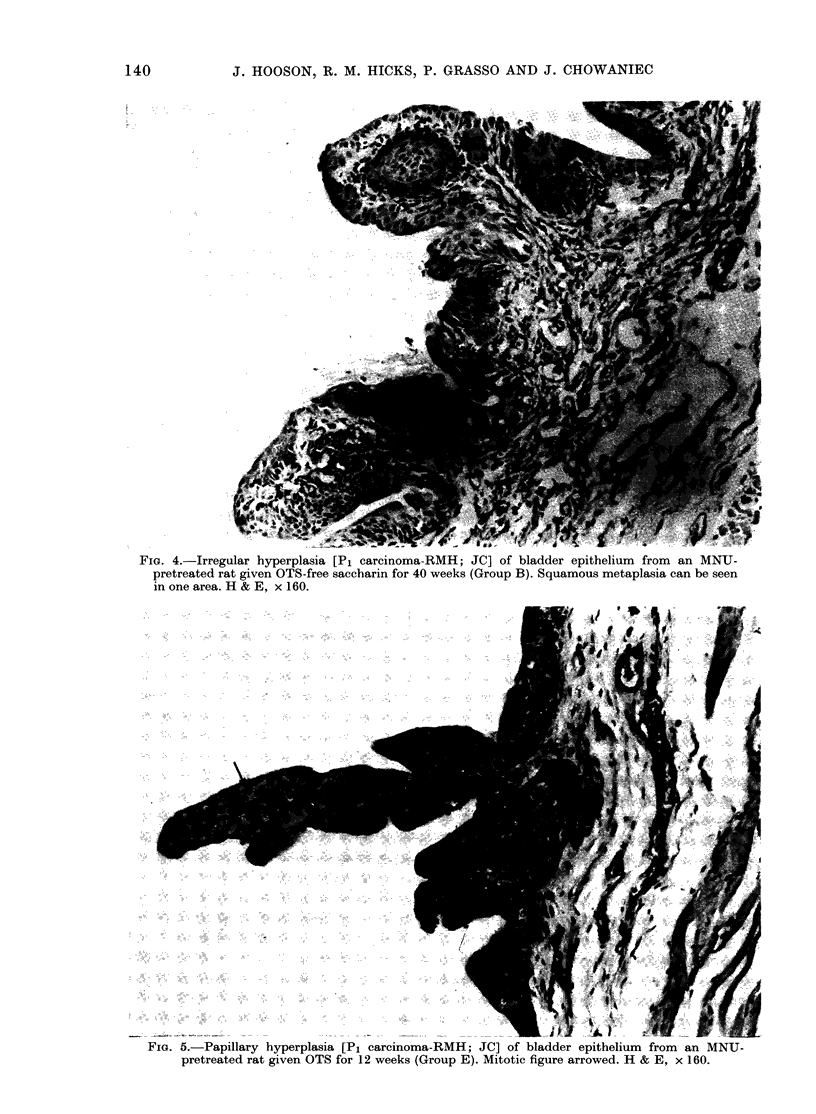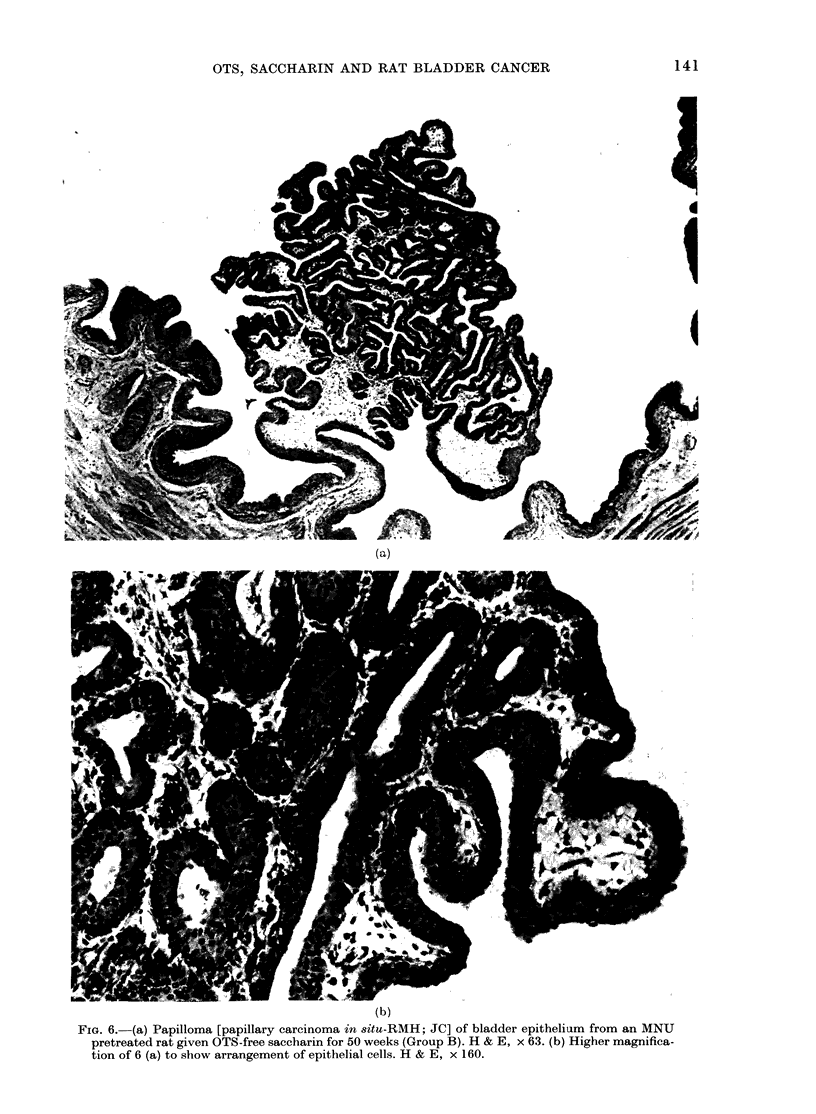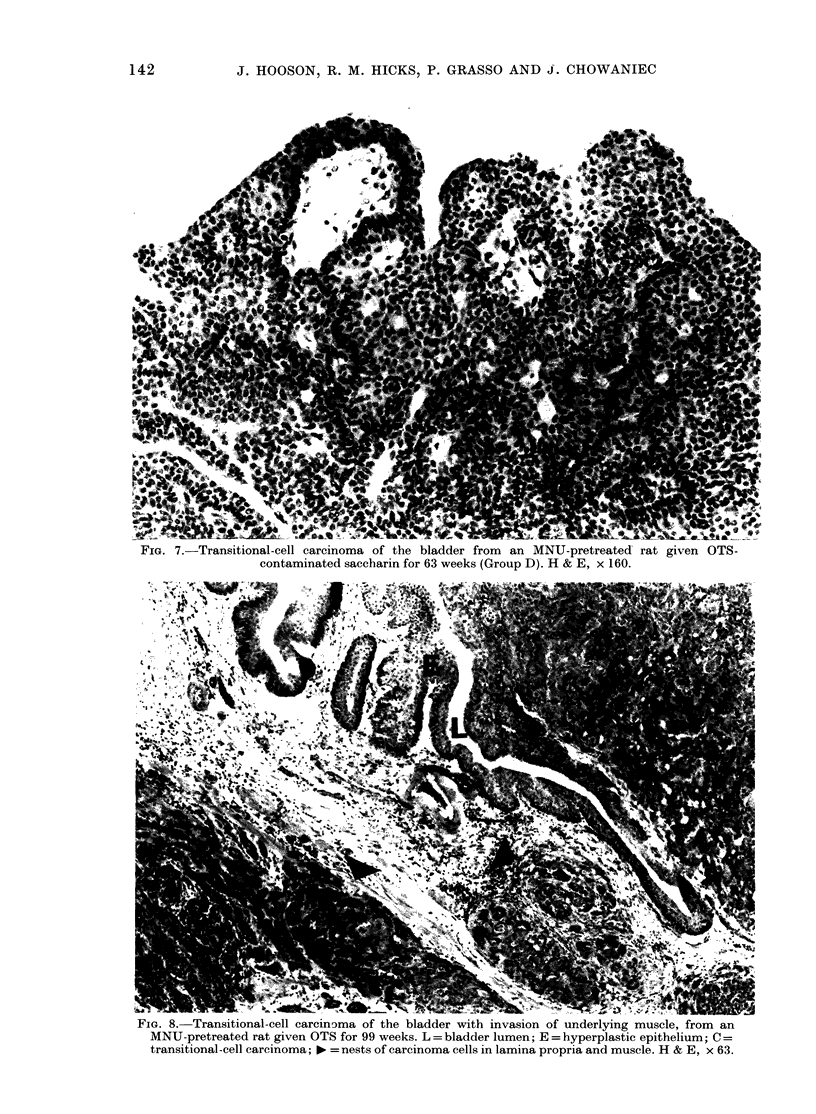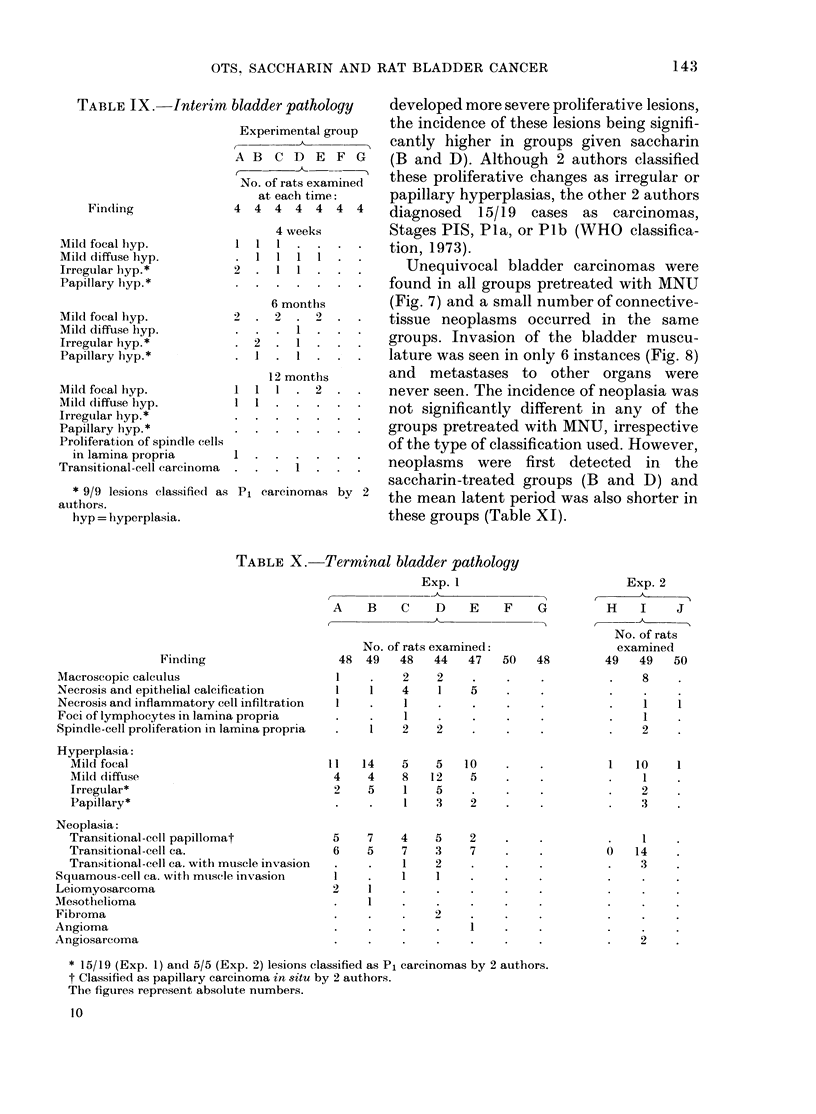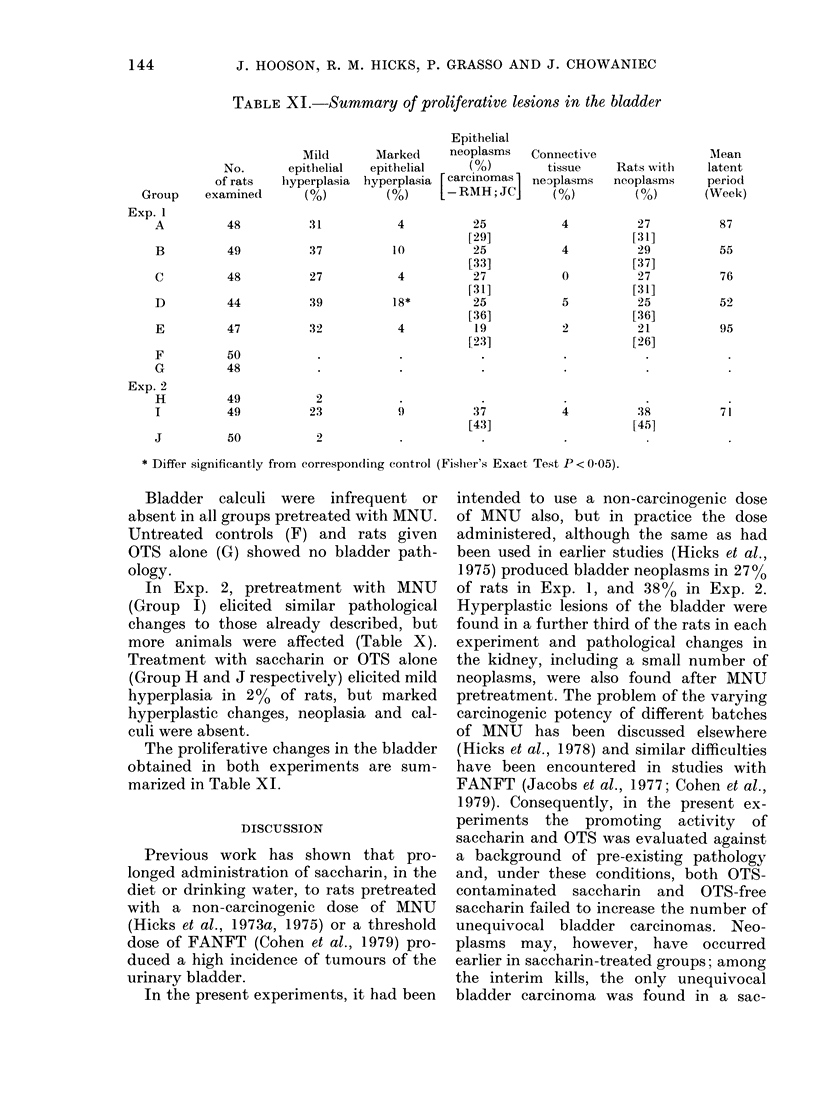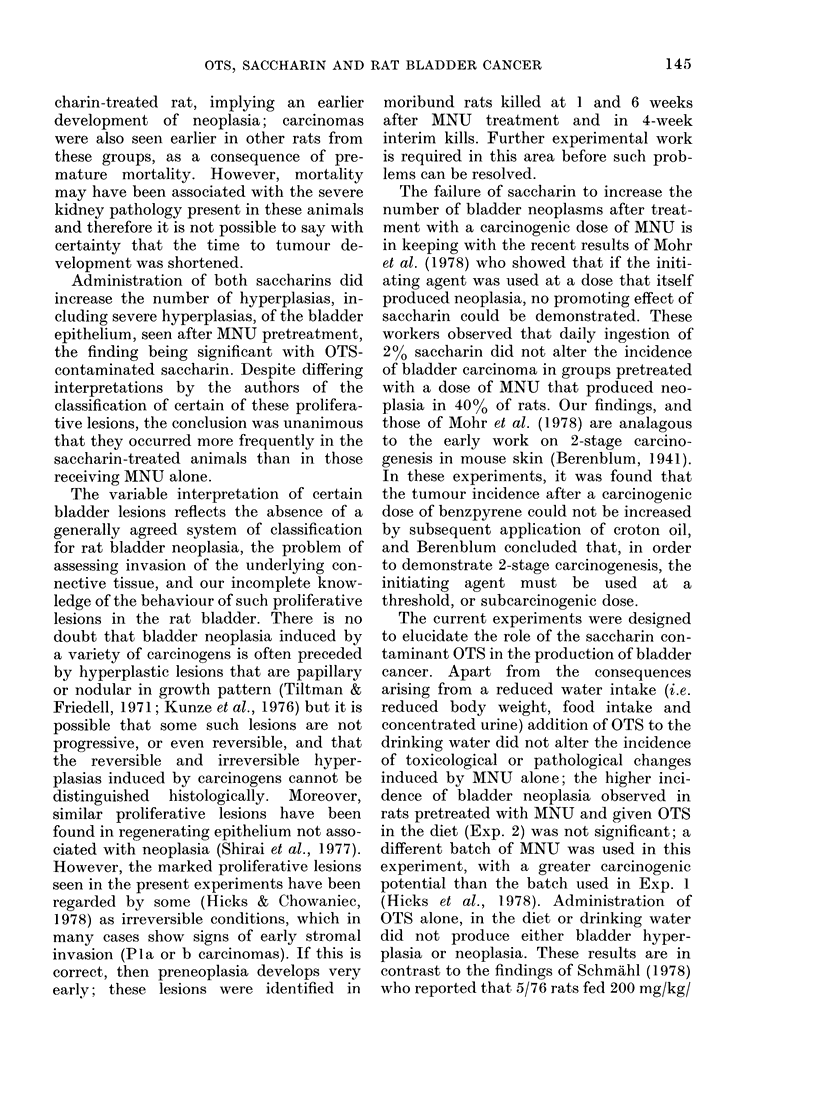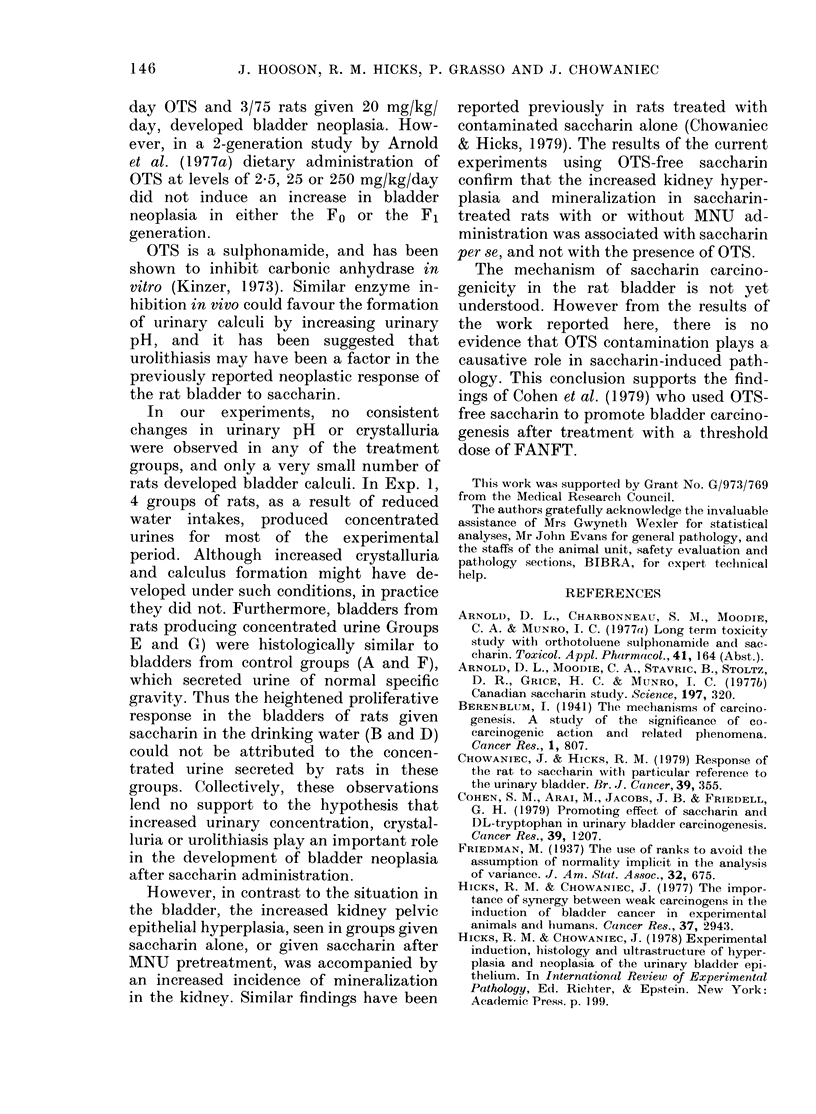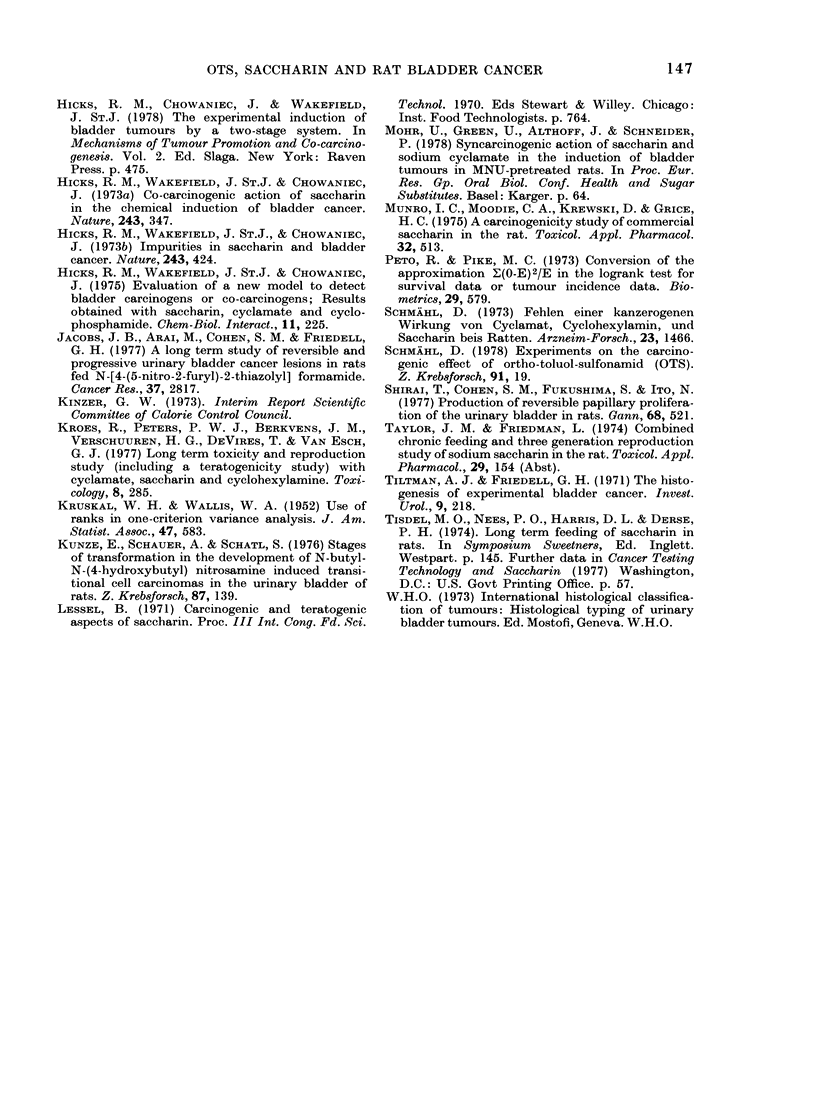# Ortho-toluene sulphonamide and saccharin in the promotion of bladder cancer in the rat.

**DOI:** 10.1038/bjc.1980.211

**Published:** 1980-07

**Authors:** J. Hooson, R. M. Hicks, P. Grasso, J. Chowaniec

## Abstract

**Images:**


*Br. J. Cancer* (1980) **42**, 129

### ORTHO-TOLUENE SULPHONAMIDE AND SACCHARIN IN THE PROMOTION OF BLADDER CANCER IN THE RAT

J. HOOSON*, R. M. HICKS†, P. GRASSO*‡ AND J. CHOWANIEC†

*From the *British Industrial Biological Research Association, Carshalton, Surrey, and the †School of Pathology, Middlesex Hospital Medical School, London*

Received 7 January 1980 Accepted 20 February 1980

**Summary.—The importance of the contaminant OTS in the promoting activity of commercial saccharin on rat bladder neoplasia was investigated. OTS, OTS-free and OTS-contaminated saccharin were administered in the drinking water or diet for 2 years to groups of rats pretreated with an intravesical instillation of MNU; OTS alone and OTS-free saccharin were also given to groups of rats not pretreated with MNU.**

**Administration of OTS was not associated with changes in urinary pH, crystalluria or calculus formation, had no effect on the histology of normal rat bladder, and did not increase the incidence of bladder hyperplasia or neoplasia elicited by pretreatment with MNU. No differences could be found between the effect of OTS-free or OTS-contaminated saccharin on bladders of rats pretreated with MNU. These results indicate that OTS contamination played no part in the reported promoting activity of saccharin on the rat bladder.**

**Administration of saccharin did not increase urinary pH, crystalluria or calculus formation, and failed to promote bladder neoplasia after a carcinogenic dose of MNU, though the numbers of proliferative lesions in the bladder were increased.**

DURING THE last 10 years, saccharin has been tested for carcinogenicity in rats, mice, hamsters and monkeys. Until recently (Arnold *et al.*, 1977*b*) single-generation feeding studies have failed to demonstrate an unequivocal carcinogenic effect of saccharin in any of these species (Lessel, 1971; Schmähl, 1973; Munro *et al.*, 1975; Kroes *et al.*, 1977; Chowaniec & Hicks, 1979). However, in 2-generation feeding studies, saccharin was found to induce bladder neoplasms in $F_1$ male rats when fed at levels of 5% or 7·5% in the diet (Taylor & Friedman, 1974; Tisdel *et al.*, 1974; Arnold *et al.*, 1977*b*). Furthermore, the administration of saccharin either in the drinking water (2 g/kg/day) or diet (4 g/kg/day) to rats previously given a single sub-carcinogenic dose of N-methyl-N-nitrosourea (MNU) produced a high incidence of bladder tumours (Hicks *et al.*, 1973*a*, 1975, 1978; Hicks & Chowaniec, 1977; Hicks *et al.*, 1978). These findings suggested that saccharin could promote carcinogenesis initiated by MNU, and recent work by Cohen *et al.* (1979) confirmed a promoting effect of saccharin on bladder neoplasia after a threshold dose of N(4-(5-nitro-2-furyl)-2-thiazolyl) formamide (FANFT).

A variety of impurities are present in commercial saccharin, the best known being ortho-toluene sulphonamide (OTS); this impurity has been detected at levels as high as 5000 pts/$10^6$ in saccharin manufactured by the Remsen-Fahlberg process, and has also been found, in much smaller amounts, in some batches of saccharin produced by the Maumée process. In the study by Hicks and her colleagues OTS was present at a concentration of 810 pts/$10^6$ (Hicks *et al.*, 1973*b*).

‡ Present address: Occupational Health Unit, B.P. Research Centre, Sunbury-on-Thames, Middlesex.

OTS is known to inhibit carbonic anhydrase (Kinzer, 1973) which could increase urinary pH, and favour the formation of urinary calculi. The possibility therefore arose that operation of such factors might have played a role in the carcinogenicity or promoting activity of saccharin.

The aim of the present experiments was therefore to examine the importance of OTS in carcinogenicity studies with saccharin, by comparing the incidence of neoplasia in MNU-pretreated bladders after long term administration of OTS-free saccharin, OTS-contaminated saccharin and OTS alone. OTS was also tested in rats not pretreated with MNU. The original experiments by Hicks and her co-workers used drinking water as the vehicle for saccharin administration, and the same vehicle was used in the main experiment of the current investigations (Exp. 1). However, some groups of rats developed signs of severe dehydration, so a second smaller experiment was set up in which compounds were administered in the diet (Exp. 2).

### MATERIALS AND METHODS

*Materials.*—MNU was synthesized at the Courtauld Institute of Biochemistry, Middlesex Hospital Medical School, and checked for purity by melting-point determination and spectrophotometric analysis. Separate samples were used for Exps 1 and 2.

Sodium saccharin, manufactured by the Maumée process and free from OTS contamination, was supplied by Sherwin Williams Chemicals, Cleveland, Ohio.

Sodium saccharin, manufactured by the Remson Fahlberg process and containing 40 pts/$10^6$ OTS, was obtained from The Boots Co. Ltd, Nottingham.

OTS was supplied by Monsanto Industrial Chemicals Co., St Louis, Missouri. The sample was found to have 1 peak on GLC analysis.

*Animals and diet.*—Female Wistar SPF weanling rats, free from the bladder parasite *Trichosomoides crassicauda*, were supplied by Oxfordshire Laboratory Animal Colonies, Bicester, Oxfordshire. Rats were housed in rooms maintained at $20 \pm 1°C$, with a relative humidity of 50–60%. Basic diet was Spratt's Laboratory Animal Diet No. 2 and drinking water was taken from the mains supply; both were available *ad libitum.*

*Experimental design.*—In Exp. 1, rats were

TABLE I.—*Experimental design*

| Group | No. of rats at start | Pre-treatment* | Treatment | Desired daily intake | Actual daily intake | Vehicle |
|---|---|---|---|---|---|---|
| | | | Exp. 1 | | | |
| A | 63 | MNU | — | — | — | — |
| B | 63 | MNU | OTS-free saccharin | 2 g/kg | 2·83 g/kg† | Drinking water |
| C | 63 | MNU | OTS | 0·08 mg/kg | 0·13 mg/kg† | Drinking water |
| D | 63 | MNU | OTS-contaminated saccharin | 2 g/kg | 3·25 g/kg† | Drinking water |
| E | 63 | MNU | OTS | 0·1% | 0·1% (70 mg/kg)† | Drinking water |
| F | 63 | — | — | — | — | — |
| G | 63 | — | OTS | 0·1% | 0·1% (70 mg/kg)† | Drinking water |
| | | | Exp. 2 | | | |
| H | 50 | — | OTS-free saccharin | 2 g/kg | 1·74 g/kg† | Diet |
| I | 50 | MNU | OTS | 70 mg/kg | 79 mg/kg† | Diet |
| J | 50 | — | OTS | 70 mg/kg | 79 mg/kg† | Diet |

* 0·15 ml of a solution containing 10 mg/ml MNU, instilled intravesicularly.

† Figures represent the mean daily intake of saccharin or OTS for each group of rats over a 2yr period, calculated from monthly data on water or food consumption, and body weight.

randomly distributed into 7 groups (A–G) each of 63 animals, and housed 7 to a cage (Table I). Groups A–E were instilled intravesically *via* urethral catheter with 0·15 ml of a freshly prepared saturated solution of MNU in 0·9% NaCl (~10 mg MNU/ml). The procedure was performed under barbiturate anaesthesia. Two weeks later, all groups were administered the appropriate chemicals in the drinking water. Group A received no chemicals, and served as the MNU-treated control. OTS-free (Group B) or OTS-contaminated (Group D) saccharin was added to drinking water to provide a desired daily intake of 2 g/kg. In practice, the concentration of saccharin in the drinking water was varied between 1·33% and 2·8% in an attempt to provide the required intake. Group C was administered OTS at a desired level of 0·08 mg/kg/day, the intake equivalent to the amount of OTS in the contaminated saccharin (40 pts/$10^6$). Groups E and G were given OTS at the level of maximum solubility in tap water (0·1%) which provided a daily intake of 70 mg/kg. Group F served as untreated controls. Administration of chemicals continued for 2 years.

Sixteen months after the start of Exp. 1, a supplementary study was initiated (Exp. 2). This consisted of 3 groups (H–J) each of 50 rats, housed 5 to a cage. The bladders of Group I rats were instilled with MNU as previously described. Eight days later, the groups were administered the appropriate chemicals in the diet. Group H received OTS-free saccharin at a desired level of 2 g/kg/day. To achieve this intake the concentration of saccharin in the diet was varied between 2 and 3·5% during the experiment. Groups I and J were fed OTS at a desired level of 70 mg/kg/day, providing a similar OTS intake to that in Groups E and G in Exp. 1. Administration of chemicals continued for 2 years.

*Measurements.*—Rats were observed daily for signs of ill health. Rats that became ill and whose condition did not improve were killed and subjected to a *post mortem* examination. Animals were weighed individually at weekly intervals; food and water consumptions were measured twice weekly, that is, over a 3-day and a subsequent 4-day period for each cage of rats. Adjustments to the concentration of the chemical in the drinking water or the diet were made, on the basis of body weight change and water and food consumptions, to maintain the correct dosage as far as possible. Three renal-function tests were carried out on the same 8–10 rats from each group at ~6-week intervals. To measure renal diluting capacity, rats were given a water load of 25 ml/kg, and urine collected over the next 2 h. The specific gravity and volume of the sample were measured, and a cell count performed. To measure renal concentrating ability, urine was collected for 6 h from rats housed without water. Samples were examined by Bili-Labstix (Ames Company, Stoke Poges, Slough) for their content of albumin, glucose, blood, bile salts, and ketones; volume and specific gravity were also measured. A second concentration test was performed on these rats by collecting urine over the last 6 h of a 24h period of water deprivation. Samples were examined for crystals, and the volume and specific gravity measured. Urine pH was measured on separately collected fresh samples.

In Exp. 1, 4 rats from each group were killed at 4, 26 and 52 weeks for interim pathological examination. Animals that died during the study were autopsied, unless this was precluded by advanced autolysis or cannibalism. Those found *in extremis*, or surviving to 102 weeks were killed by exsanguination from the abdominal aorta under barbiturate anaesthesia. The kidneys were weighed, and fixed in 10% buffered formalin. The presence of any macroscopic calculi in the urinary tract was noted. The bladders were gently distended by injection of fixative, opened and examined for gross lesions. All other tissues appearing abnormal at post mortem were preserved in formalin. After fixation, the bladders were cut transversely into 3 pieces. The right kidney was cut transversely and the left longitudinally. All tissues were embedded in paraffin wax, sectioned and stained with haematoxylin and eosin.

*Statistical analyses.*—In Exp. 1, Group F was compared with Groups A and G, and Group A with Groups B, C, D and E. In Exp. 2, Groups H, I and J were compared with each other. Cumulative mortality was analysed by the method of Peto & Pike (1973); differences in body weights and urine analyses were examined by Student's $t$ test; food and water consumption were analysed by Friedman's test (1937) and by the Kruskal & Wallis test (1952). Kidney weights and kidney and bladder pathology were compared by $\chi^2$ or Fisher Exact Test.

### RESULTS

### *General observations, mortality and weight gain*

A proportion of rats ingesting either OTS-free or OTS-contaminated saccharin in the drinking water (Groups B and D) developed a mild diarrhoea that persisted throughout the study. Otherwise the behaviour and appearance of rats in all groups in Exps 1 and 2 were normal.

Although in Exp. 1 there was a tendency for rats in all groups treated with MNU (A–E) to die earlier than rats not receiving MNU (F and G) cumulative mortality was significantly increased only in Groups B and D, which were given saccharin in the drinking water (Table II). In these 2 groups, only 2 and 7 rats respectively were still alive at 96 weeks. No significant differences in mortality were seen in Groups H–J in Exp. 2.

TABLE II.—*Cumulative mortality*

| Week on test | Total no. of deaths | | | | | | | | | |
|---|---|---|---|---|---|---|---|---|---|---|
| | Exp. 1 | | | | | | | Exp. 2 | | |
| | Group: | | | | | | | Group: | | |
| | A | B | C | D | E | F | G | H | I | J |
| 1 | 1 | 0 | 0 | 0 | 0 | 0 | 0 | 0 | 0 | 0 |
| 9 | 1 | 2 | 0 | 1 | 0 | 0 | 0 | 0 | 1 | 0 |
| 17 | 1 | 3 | 0 | 4 | 1 | 0 | 0 | 0 | 2 | 0 |
| 25 | 1 | 3 | 0 | 4 | 2 | 0 | 0 | 0 | 2 | 0 |
| 36 | 3 | 11 | 3 | 7 | 3 | 1 | 1 | 0 | 4 | 0 |
| 43 | 6 | 17 | 3 | 11 | 4 | 2 | 1 | 1 | 6 | 0 |
| 52 | 10 | 22 | 7 | 16 | 5 | 2 | 2 | 1 | 6 | 1 |
| 60 | 12 | 27 | 8 | 19 | 6 | 2 | 2 | 4 | 7 | 3 |
| 68 | 12 | 32 | 10 | 23 | 7 | 5 | 2 | 4 | 11 | 5 |
| 76 | 16 | 35 | 11 | 28 | 11 | 8 | 5 | 5 | 17 | 6 |
| 84 | 22 | 43 | 16 | 37 | 16 | 13 | 12 | 13 | 22 | 11 |
| 96 | 34 | 49 | 25 | 44 | 28 | 24 | 22 | 25 | 27 | 20 |
| Trend | | 23·34*** | | 14·46*** | | | | | | |

Figures are the total number of animals dead or killed *in extremis* from groups of 51 (A–G) or 50 H–J).

Groups marked with asterisks differ significantly from their appropriate controls (Peto & Pike, 1973).

***$P < 0·001$.

Rats in Groups E and G weighed less than their relevant controls at the start of Exp. 1 and the differences had increased by 9 weeks (Table III). By Week 25, the groups given saccharin in the drinking water (B and D) had also significantly smaller body-weight gains than the relevant controls, and the reduced body-weight gains in these 4 groups persisted throughout the experiment. In Exp. 2, no differences in body-weight were seen between rats receiving OTS with or without MNU, despite a slightly higher initial body weight in Group J. Rats from the saccharin-treated Group H tended to have slightly higher body weights than those in Groups I or J, but the differences were only statistically significant intermittently.

### *Food and water consumptions*

In Exp. 1, food consumption showed no consistent differences between the groups, with some groups having occasionally slightly higher or lower consumptions than corresponding controls. However, when the data were analysed on a cumulative basis between 4, 13, 41 and 92 weeks (Friedman, 1937) both the saccharin-treated groups (B and D) and the high-dose OTS group (E) showed reduced consumption (Table IV).

In Exp. 2, occasional differences in food consumptions were also seen between the 3 groups, the cumulative food consumption being significantly higher in Group H.

Water consumption was decreased in groups receiving saccharin (B and D) or high-dose OTS (E and G) after 5 weeks of treatment in Exp. 1 and, by 13 weeks, the differences were statistically significant in all 4 groups. Consumption remained low in Groups E and G for the duration of the experiment, but in Group B, water intake had returned to control levels by 52 weeks, whereas Group D had raised consumption for the last year of the experiment. In Exp. 2, the saccharin treated Group H had a significantly higher cumulative water intake than Groups I and J.

The calculated daily intakes of saccharin and OTS in all groups are given in Tables I and V. The desired intake of saccharin in Groups B, D and H was 2 g/kg/day. However, because of fluctuations in food and water consumption,

TABLE III.—*Mean body weights*

| Week on test | Body weight (g) Exp. 1 Group: A | B | C | D | E | F | G | Exp. 2 Group: H | I | J |
|---|---|---|---|---|---|---|---|---|---|---|
| 0 | 189 | 189 | 188 | 188 | 183 | 189 | 181** | 144 | 144 | 150* |
| 1 | 233*** | 232* | 232 | 235* | 233 | 240 | 224*** | 187* | 181 | 186 |
| 5 | 248*** | 251 | 254 | 253 | 239* | 261 | 242*** | 218* | 211 | 212 |
| 9 | 266* | 267 | 272 | 271 | 250** | 276 | 254*** | 231 | 230 | 232 |
| 13 | 277* | 268* | 281 | 268* | 261*** | 287 | 261*** | 248 | 243 | 243 |
| 17 | 283* | 271* | 289 | 278 | 269** | 296 | 271*** | 261 | 255 | 256 |
| 25 | 296** | 271*** | 304 | 276*** | 277*** | 312 | 279*** | 278 | 270 | 273 |
| 39 | 323 | 276*** | 324 | 278*** | 296*** | 332 | 300*** | 303* | 290 | 293 |
| 52 | 354 | 282*** | 356 | 297*** | 318*** | 373 | 317*** | 325* | 311 | 310 |
| 68 | 364 | 293*** | 371 | 301*** | 329*** | 391 | 330*** | 352 | 335 | 356 |
| 85 | 356 | 273*** | 373 | 275*** | 326* | 384 | 331*** | 358 | 349 | 350 |
| 97 | 340 | 236* | 355 | 305 | 319 | 362 | 332* | 353 | 345 | 348 |

Figures are the mean for all survivors in each group. Those marked with asterisks differ significantly from their appropriate controls (Student's *t* test).
* $P<0{\cdot}05$; ** $P<0{\cdot}01$; *** $P<0{\cdot}001$.

TABLE IV.—*Food and water consumption*

| Week on test | Exp. 1 Group: A | B | C | D | E | F | G | Exp. 2 Group: H | I | J |
|---|---|---|---|---|---|---|---|---|---|---|
| | | | | *Food consumption (g/rat/day)*† | | | | | | |
| 0 | 17·8 | 19·0 | 19·6 | 20·0 | 19·2 | 19·3 | 18·9 | 16·8 | 17·1 | 16·6 |
| 4 | 19·8 | 19·1 | 19·5 | 19·8 | 17·1 | 18·8 | 17·7 | 17·4 | 17·5 | 16·3 |
| 9 | 17·8 | 15·6 | 17·0 | 16·1 | 15·6 | 16·8 | 15·7 | 18·1 | 18·4 | 18·8 |
| 13 | 14·5 | 12·6* | 14·3 | 13·9** | 14·7** | 14·6 | 14·7 | 18·7 | 17·2 | 16·8 |
| 24 | 17·7 | 16·2 | 17·6 | 15·1 | 14·8 | 16·3 | 14·9 | 16·9 | 15·9 | 15·4 |
| 41 | 18·5 | 16·5** | 17·4 | 15·3* | 16·5* | 15·4 | 15·9 | 19·0** | 17·5 | 17·0 |
| 52 | 16·7 | 14·9 | 16·6 | 15·8 | 16·0 | 16·5 | 16·0 | 18·5 | 17·8 | 18·5 |
| 68 | 17·0 | 13·9 | 16·0 | 15·0 | 15·5 | 16·1 | 14·4 | 16·8 | 14·5 | 15·2 |
| 84 | 22·0 | 24·7 | 19·3 | 17·0 | 19·0 | 19·1 | 21·6 | 18·6 | 16·6 | 16·3 |
| 92 | 17·6 | 9·2** | 18·2 | 15·6* | 17·6* | 17·5 | 17·5 | 17·5* | 16·4 | 16·3 |
| | | | | *Water consumption (ml/rat/dat)*‡ | | | | | | |
| 0 | 27·4 | 30·1 | 36·5 | 35·6 | 34·0 | 34·3 | 36·6 | 33·6 | 35·9 | 30·8 |
| 5 | 34·9 | 27·6 | 34·4 | 30·3 | 25·1 | 40·1 | 24·2 | 33·4 | 34·9 | 28·0 |
| 9 | 34·2 | 26·1 | 38·9 | 26·8 | 20·3* | 37·8 | 22·1** | 39·6 | 38·2 | 33·5 |
| 13 | 34·0 | 15·8** | 33·7 | 17·4** | 19·8* | 36·6 | 18·8* | 35·5 | 37·5 | 32·8 |
| 24 | 25·3 | 19·9 | 34·7 | 21·4 | 17·1* | 36·4 | 16·9** | 35·4 | 37·7 | 32·1 |
| 41 | 30·3 | 20·4* | 32·6 | 23·0** | 20·8* | 28·0 | 18·8** | 40·7* | 38·6 | 30·3 |
| 52 | 30·8 | 32·6 | 29·9 | 37·8 | 20·6** | 30·9 | 19·1* | 39·6* | 31·5 | 30·8 |
| 68 | 31·7 | 33·6 | 33·7 | 37·9 | 23·2 | 31·8 | 19·2* | 45·8* | 33·4 | 30·6 |
| 84 | 33·2 | 29·6 | 38·9 | 51·6 | 24·1 | 31·7 | 23·5 | 54·2 | 33·8 | 32·4 |
| 92 | 34·0 | 25·8 | 36·9 | 53·0 | 35·3 | 37·5 | 27·8* | 50·1* | 39·1 | 28·8 |

Food and water consumption figures are means for 9 cages each containing initially 7 rats in Groups A–G and for 10 cages each containing initially 5 rats, in Groups H–J. Those marked with asterisks differ significantly from their appropriate controls.
† Friedman's test.
‡ Kruskal and Wallis test.
* $P<0{\cdot}05$; ** $P<0{\cdot}01$.

TABLE V.—*Calculated intake of OTS and saccharin*

| | Compound intake (g/kg/day) | | | | | | | | | | |
|---|---|---|---|---|---|---|---|---|---|---|---|
| | Exp. 1 | | | | | | | | Exp. 2 | | |
| Week on test | Group: A | B | C | D | E | F | G | Week on test | Group: H | I | J |
| 0 | 0 | 0 | 0 | 0 | 0 | 0 | 0 | 0 | 0 | 0 | 0 |
| 1 | 0 | 3·20 | 0·07* | 3·56 | 0·08 | 0 | 0·10 | 1 | 1·80 | 0·10 | 0·09 |
| 4 | 0 | 2·93 | 0·07 | 2·71 | 0·08 | 0 | 0·13 | 5 | 1·58 | 0·08 | 0·08 |
| 9 | 0 | 0·98 | 0·10 | 0·99 | 0·08 | 0 | 0·09 | 9 | 1·56 | 0·08 | 0·08 |
| 13 | 0 | 1·28 | 0·07 | 1·40 | 0·08 | 0 | 0·07 | 13 | 1·51 | 0·07 | 0·07 |
| 16 | 0 | 2·27 | 0·07 | 2·43 | 0·06 | 0 | 0·09 | 16 | 1·76 | 0·08 | 0·09 |
| 24 | 0 | 2·11 | 0·06 | 2·20 | 0·06 | 0 | 0·06 | 25 | 1·53 | 0·07 | 0·07 |
| 41 | 0 | 2·06 | 0·06 | 2·74 | 0·07 | 0 | 0·06 | 42 | 1·95 | 0·09 | 0·08 |
| 52 | 0 | 3·31 | 0·19 | 3·62 | 0·07 | 0 | 0·06 | 51 | 1·82 | 0·08 | 0·09 |
| 68 | 0 | 3·26 | 0·20 | 3·58 | 0·07 | 0 | 0·06 | 68 | 1·67 | 0·07 | 0·07 |
| 84 | 0 | 3·15 | 0·24 | 5·28 | 0·08 | 0 | 0·07 | 85 | 1·81 | 0·08 | 0·08 |
| 92 | 0 | 3·13 | 0·22 | 5·17 | 0·12 | 0 | 0·08 | 98 | 1·70 | 0·08 | 0·08 |

* Compound intake in Group C expressed as mg/kg/day.
The values for compound intake are calculated from data on water consumption and body weight in Exp. 1 and from data on food consumption and body weight in Exp. 2.

Groups B and D received rather less than this amount for the first 4 months of the study, and more during the last 12 months. Group H received slightly less than the desired intake throughout the study.

*Urine studies and kidney weights*

In Exps 1 and 2, some rats from all groups pretreated with MNU (A–E and I) had erythrocytes, acute inflammatory cells and cell debris in the urine when examined 2 weeks after MNU treatment. Intermittent haematuria was found in these groups throughout the experiments. Semi-quantitative tests for glucose, ketones, bile salts and albumin showed no marked differences between any groups. Crystalluria was observed in all groups, becoming more apparent after the first 6 months of treatment. The range of pH measured on fresh urine from individual rats varied from 5–8. With time, there was a tendency for more samples to have an acid pH, but this occurred in all groups and could not be related to treatment. Measurements of cell excretion were similar in all groups (Table VI).

In Exp. 1, concentration/dilution tests performed during the first 12 months showed higher urinary specific gravity and smaller volume from rats in Groups B, D, E and G than in appropriate controls (Table VI). In the concentration tests, the most statistically significant difference was observed in these 4 groups when specific gravities were compared on the 0–6h sample. In the dilution test, Groups B and D consistently showed highly significant differences of both urinary specific gravity and volume, whereas similar changes in Groups E and G were only statistically significant intermittently. The pattern of changes seen in the dilution test persisted throughout the experiment but, in the concentration test, the differences became less marked with time.

At 50 weeks, a further observation was recorded in Groups B and D, when decreased specific gravities occurred in the 18–24h sample. Similar findings were observed for the remainder of the experiment, but the decreases were not always statistically significant.

In Exp. 2, concentration tests indicated a significant decrease in specific gravity of urine from rats pretreated with MNU (Group I) when 18–24h samples were analysed at 2, 7 and 11 weeks. Concentration tests performed during the rest of the experiment, and dilution tests throughout the experiment, showed no consistent differences between the groups.

Absolute kidney weights were lower in Groups B, D and G than in the relevant controls (Table VIII). Expressed relative to body weight, Groups B, D, E and G had

TABLE VI.—*Renal concentration and dilution tests and urinary cell excretion rates*

| Group | Cell excretion ($10^3$/h) | Concentration test: Sp. gr. 0–6 h | Concentration test: Sp. gr. 18–24 h | Concentration test: Volume (ml) 0–6 h | Concentration test: Volume (ml) 18–24 h | Dilution test (2 h): Sp. gr. | Dilution test (2 h): Volume (ml) |
|---|---|---|---|---|---|---|---|
| | | | Exp. 1 | | | | |
| | | | Week 13 | | | | |
| A | 7 | 1·038 | 1·068 | 1·3 | 0·7 | 1·005 | 3·5 |
| B | 1 | 1·082*** | 1·077 | 0·4 | 0·7 | 1·038* | 1·1* |
| C | 1 | 1·037 | 1·049 | 2·1 | 1·5 | 1·007 | 4·0 |
| D | 2 | 1·070*** | 1·072 | 0·9 | 0·9 | 1·016* | 2·4 |
| E | 1 | 1·060*** | 1·069 | 1·0 | 0·8 | 1·012* | 2·7 |
| F | 3 | 1·029 | 1·073 | 1·1 | 0·4 | 1·005 | 4·5 |
| G | 3 | 1·060** | 1·065 | 0·8 | 0·9 | 1·008 | 2·6 |
| | | | Week 25 | | | | |
| A | 1 | 1·035 | 1·072 | 2·4 | 0·6 | 1·010 | 3·6 |
| B | 1 | 1·071*** | 1·070 | 1·0* | 0·6 | 1·071*** | 0·3** |
| C | 2 | 1·033 | 1·055 | 2·9 | 1·3 | 1·006 | 3·9 |
| D | 0 | 1·075*** | 1·071 | 1·7 | 1·1 | 1·056** | 1·1 |
| E | 1 | 1·059** | 1·073 | 1·2 | 0·7 | 1·014 | 2·2 |
| F | 1 | 1·033 | 1·081 | 1·3 | 0·4 | 1·003 | 5·2 |
| G | 1 | 1·071 | 1·086 | 0·9 | 0·2* | 1·019* | 2·4*** |
| | | | Week 36 | | | | |
| A | 1 | 1·039 | 1·075 | 1·6* | 0·6 | 1·005 | 5·8 |
| B | 1 | 1·064*** | 1·068 | 2·0 | 0·7 | 1·045*** | 0·7*** |
| C | 0 | 1·039 | 1·066 | 2·3 | 0·8 | 1·004 | 6·1 |
| D | 0 | 1·067** | 1·064 | 2·0 | 0·9 | 1·052*** | 0·6*** |
| E | 1 | 1·066** | 1·070 | 0·9* | 0·5 | 1·013* | 2·8* |
| F | 1 | 1·044 | 1·076 | 2·5 | 0·5 | 1·005 | 5·6 |
| G | 0 | 1·066*** | 1·086 | 1·3* | 0·8* | 1·011 | 3·1* |
| | | | Week 55 | | | | |
| A | 3 | 1·044 | 1·079 | 1·3 | 0·6 | 1·004 | 5·4 |
| B | 1 | 1·063* | 1·060* | 1·8 | 0·7 | 1·046*** | 1·1*** |
| C | 2 | 1·035 | 1·074 | 2·0 | 0·6 | 1·005 | 5·9 |
| D | 3 | 1·055 | 1·046*** | 2·8* | 1·2 | 1·041** | 0·8*** |
| E | 3 | 1·066* | 1·080 | 1·1 | 0·6 | 1·009* | 2·9* |
| F | 2 | 1·040 | 1·082 | 2·1 | 0·5 | 1·004 | 6·6 |
| G | 3 | 1·073*** | 1·093 | 0·8*** | 0·7 | 1·008* | 1·8*** |
| | | | Exp. 2 | | | | |
| | | | Week 2 | | | | |
| H | 1 | 1·041 | 1·074 | 2·3 | 0·7 | 1·007 | 2·7 |
| I | 1 | 1·026 | 1·059*** | 1·2 | 0·8 | 1·006 | 2·1 |
| J | 0 | 1·031 | 1·073 | 1·3 | 0·6 | 1·006 | 2·8 |
| | | | Week 11 | | | | |
| H | 1 | 1·051 | 1·084 | 1·3 | 0·7 | 1·009 | 3·3 |
| I | 1 | 1·042 | 1·067** | 1·5 | 0·6 | 1·010 | 3·1 |
| J | 1 | 1·038 | 1·072 | 2·1 | 0·6 | 1·006 | 4·1 |
| | | | Week 37 | | | | |
| H | 1 | 1·051 | 1·076 | 1·6 | 0·7 | 1·006 | 4·9 |
| I | 1 | 1·054 | 1·073 | 1·2 | 0·6 | 1·007 | 4·1 |
| J | 1 | 1·035 | 1·065 | 1·7 | 0·6 | 1·004 | 5·3 |
| | | | Week 75 | | | | |
| H | 1 | 1·033 | 1·063 | 3·4 | 1·3 | 1·006 | 5·4 |
| I | 1 | 1·037 | 1·066 | 2·7 | 1·1 | 1·004 | 6·8 |
| J | 1 | 1·037 | 1·079 | 2·9 | 0·9 | 1·004 | 6·4 |

The figures are means for groups of 7 (A–G) or 10 (H–J) rats. Those marked with asterisks differ significantly from their appropriate controls. (Student's *t* test).
* $P < 0{\cdot}05$; ** $P < 0{\cdot}01$; *** $P < 0{\cdot}001$.

TABLE VII.—*Incidence of neoplasia in tissues other than kidney and bladder*

| | Exp. 1 | | | | | | | Exp. 2 | | |
|---|---|---|---|---|---|---|---|---|---|---|
| Tissue | A | B | C | D | E | F | G | H | I | J |
| Adrenal | | | | | | | | | | |
| Pheochromocytoma | . | . | . | . | . | . | 2 | 1 | . | 1 |
| Brain | | | | | | | | | | |
| Glioma | . | . | . | . | . | . | 1 | . | . | . |
| Gastro-intestinal tract | | | | | | | | | | |
| (a) *Stomach* | | | | | | | | | | |
| Carcinoma | . | . | . | . | . | . | . | 1 | . | . |
| Leiomyosarcoma | . | . | . | . | 1 | . | . | . | . | . |
| (b) *Ileum* | | | | | | | | | | |
| Adenocarcinoma | . | 1 | . | . | . | . | . | . | . | . |
| Histiocytoma | . | . | 1 | . | . | . | . | . | . | . |
| Reticulum cell sarcoma | . | . | . | . | . | . | . | . | . | 1 |
| Liver | | | | | | | | | | |
| Cholangioma | . | . | . | . | . | . | . | 1 | . | 1 |
| Hepatocellular carcinoma | . | . | . | . | . | 1 | . | . | . | . |
| Reticulum cell sarcoma | . | . | . | . | 1 | . | . | . | . | 1 |
| Kupffer cell sarcoma | . | . | . | . | . | . | . | . | 1 | . |
| Lung | | | | | | | | | | |
| Adenoma | . | . | . | . | . | . | 1 | . | . | . |
| Metastasis (from liver) | . | . | . | . | 1 | . | . | . | 1 | 1 |
| Lymph nodes | | | | | | | | | | |
| Lymphosarcoma | . | . | . | 1 | . | . | . | . | . | . |
| Haemangioma | . | . | . | . | . | . | 1 | . | . | 1 |
| Angiosarcoma | . | . | 1 | . | . | . | . | . | . | . |
| Mammary gland | | | | | | | | | | |
| Adenoma | 3 | . | . | . | . | . | 1 | 2 | 1 | . |
| Fibroadenoma | 2 | . | 4 | . | 1 | 1 | 3 | 4 | . | 4 |
| Adenocarcinoma | 1 | . | 1 | . | 2 | 2 | 1 | . | 1 | 1 |
| Ovary | | | | | | | | | | |
| Granulosa-cell tumour | . | . | 1 | . | . | 2 | 1 | . | . | 1 |
| Pancreas | | | | | | | | | | |
| Islet-cell adenoma | . | . | . | . | . | 1 | . | 1 | . | . |
| Peritoneum | | | | | | | | | | |
| Angiosarcoma | . | . | 1 | . | . | . | . | . | . | . |
| Pituitary | | | | | | | | | | |
| Adenoma | 28 | 8 | 24 | 8 | 28 | 36 | 29 | 32 | 25 | 32 |
| Skin & s.c. tissue | | | | | | | | | | |
| Lipoma | 1 | . | . | . | . | . | . | . | . | . |
| Fibroma | . | . | . | . | . | . | . | . | . | 1 |
| Neurofibroma | 1 | . | . | . | . | . | . | . | . | . |
| Sarcoma | . | 1 | . | . | . | . | . | . | . | 1 |
| Fibrosarcoma | . | . | . | . | . | 2 | . | . | . | . |
| Lymphosarcoma | . | . | . | . | . | . | 1 | . | . | . |
| Basal-cell carcinoma | . | . | 1 | . | . | . | 1 | . | 1 | . |
| Squamous-cell carcinoma | . | . | . | . | . | . | . | 1 | . | . |
| Spleen | | | | | | | | | | |
| Myeloid leukaemia | . | . | . | . | . | . | . | . | . | 1 |
| Thymus | | | | | | | | | | |
| Lymphosarcoma | . | . | 2 | . | . | 2 | 2 | 2 | 1 | 1 |
| Thyroid | | | | | | | | | | |
| Adenoma | 1 | . | . | . | . | . | . | . | . | . |
| Adenocarcinoma | 2 | . | . | . | 1 | . | . | . | . | . |
| C-cell carcinoma | . | . | 1 | . | . | . | . | . | . | . |
| Uterus | | | | | | | | | | |
| Polyp | . | . | 1 | 2 | 1 | 3 | 2 | 3 | 1 | 7 |
| Fibroma | 1 | . | . | . | . | 2 | . | . | . | . |
| Adenocarcinoma | . | . | . | 1 | . | . | . | 1 | . | . |
| Sarcoma | 2 | . | . | 1 | . | 1 | . | . | . | . |
| Leiomyosarcoma | . | . | . | . | 1 | . | . | . | . | . |
| Pericytoma | . | 1 | . | . | . | . | . | . | . | . |
| Haemangiosarcoma | . | . | . | . | . | . | . | 1 | . | . |
| Reticulum-cell sarcoma | . | . | . | . | . | 1 | . | . | . | . |

TABLE VIII.—*Terminal kidney weights and kidney pathology*

| | Exp. 1 | | | | | | | Exp. 2 | | |
|---|---|---|---|---|---|---|---|---|---|---|
| | A | B | C | D | E | F | G | H | I | J |
| | No. of rats examined: | | | | | | | No. of rats examined: | | |
| Feature | 48 | 49 | 48 | 44 | 47 | 50 | 48 | 50 | 49 | 50 |
| *Weights* | | | | | | | | | | |
| Absolute weight (g) | 2·59 | 2·32* | 2·51 | 2·21** | 2·58 | 2·49 | 2·41* | 2·64 | 2·43 | 2·30 |
| Relative weight (g/kg bw) | 0·85 | 0·98* | 0·78 | 0·96* | 0·94* | 0·75 | 0·80 | 0·86 | 0·82 | 0·72 |
| *Pathology* | | | | | | | | | | |
| Glomerulonephrosis | 35 | 34 | 40 | 27 | 41 | 49 | 48 | 45 | 36 | 43 |
| Pyelonephritis | 4 | 1 | 4 | 5 | 1 | 0 | 0 | 0 | 6 | 1 |
| Hydronephrosis | 4 | 0 | 4 | 3 | 1 | 2 | 0 | 3 | 10 | 1 |
| Pelvic epithelial hyperplasia | 13 | 24* | 16 | 29*** | 13 | 0 | 0 | 43*** | 19 | 21 |
| Mineral deposits: | | | | | | | | | | |
| Macroscopic calculi | 1 | 1 | 3 | 1 | 0 | 0 | 0 | 3 | 4 | 1 |
| Cortico-medullary | 1 | 7 | 0 | 4 | 0 | 0 | 0 | 8 | 6 | 8 |
| Other | 4 | 14*** | 11 | 19*** | 9 | 4 | 7 | 44*** | 19 | 24 |
| Neoplasia: | | | | | | | | | | |
| Transitional cell carcinoma | 0 | 1 | 1 | 1 | 3 | 0 | 0 | 0 | 2 | 0 |
| Adenocarcinoma | 0 | 1 | 0 | 0 | 0 | 0 | 0 | 0 | 0 | 0 |
| Undifferentiated carcinoma | 0 | 0 | 0 | 0 | 0 | 0 | 0 | 0 | 0 | 1 |
| Angioma | 0 | 0 | 0 | 1 | 0 | 0 | 0 | 0 | 0 | 0 |
| Hamartoma | 1 | 1 | 0 | 0 | 0 | 0 | 0 | 0 | 0 | 0 |

The figures represent the incidence of findings amongst the number of rats shown. Those marked with asterisks differ significantly ($\chi^2$ or Fisher's Exact Test) from their appropriate controls.
* $P < 0·05$; ** $P < 0·01$; *** $P$ 0·001.

increased kidney weights. In Exp. 2, the saccharin-treated Group H showed the highest absolute and relative kidney weights and Group J the lowest.

*Pathology*

A wide variety of non-neoplastic and neoplastic pathology was seen in all groups in both experiments. The changes were similar to those reported in ageing rats, and no treatment-related pathology could be determined in organs other than kidney and bladder. Tumours arising away from the urinary tract are summarized in Table VII. The relatively small number of pituitary tumours in Groups B and D was consistent with the early mortality in these groups.

*Kidney pathology.*—Glomerulonephrotic changes, consistent with ageing, were seen in all groups (Table VIII); the lower incidence of severe glomerulonephrosis seen in Groups B and D again reflected the early mortality in the MNU and saccharin-treated rats. Pyelonephritis occurred in some rats from all groups pretreated with MNU (A–E and I).

In Exp. 1, only MNU-pretreated groups showed hyperplasia of the pelvic epithelium (Fig. 1) the incidence being significantly higher after administration of saccharin (Groups B and D). Microscopic calcium deposits were present in some rats from all groups (A–G) but again with a significantly higher incidence in Groups B and D. Calcification occurred in the collecting ducts, the pelvic epithelium, and the pelvic space, as well as in the area of the cortico–medullary junction. The few macroscopic calculi recorded were associated with MNU treatment.

In Exp. 2, 40% of rats in both Groups I and J and 80% of rats fed saccharin alone (Group H) developed pelvic epithelial hyperplasia. Microscopic calcification was observed in all 3 groups, but again the highest frequency (90%) was in Group H. Macroscopic calculi occurred infrequently in all groups.

Small numbers of kidney neoplasms,

FIG. 1.—Marked hyperplasia of renal pelvic epithelium in the kidney of an MNU-pretreated rat given OTS for 12 weeks (Group E). Inflammatory-cell debris (D) is present in the pelvic space (PS). HPE = hyperplastic pelvic epithelium. H & E, × 100.

mostly transitional cell carcinomas, were found to be associated with MNU treatment but, in addition, one undifferentiated carcinoma was also seen in Group J.

*Bladder pathology*.—Hyperplastic lesions of the bladder were classified as mild focal, mild diffuse, irregular or papillary. Mild hyperplasias were characterized by an increased number of epithelial cell layers, which remained regular and well differentiated (Fig. 2). If more than 20% of the epithelium was hyperplastic, the lesion was termed diffuse. Irregular hyperplasias were basically endophytic in growth pattern, and were characterized by broad-front extensions into the lamina propria, including Von Brunn's nests (Figs 3 and 4). Papillary hyperplasias were exophytic lesions, consisting of fingerlike projections into the bladder lumen of well differentiated transitional epithelium, surrounding a thin fibro-vascular stroma (Fig. 5). Neoplasms were diagnosed as malignant on the basis of loss of differentiation, cellular atypia, nuclear pleomorphism, number of mitoses and invasion. Inevitably, these judgements were subjective in certain cases, and consequently some exophytic neoplasms were diagnosed as benign papillomas by 2 authors (JH; PG) and as papillary carcinoma *in situ* (PIS, WHO classification, 1973) by 2 authors (RMH; JC) (Figs 6a,b).

The bladder lesions observed at interim kills at 1, 6 and 12 months in Exp. 1 are shown in Table IX. One unequivocal neoplasm was seen at 12 months in Group D.

Terminal bladder pathology for all groups is illustrated in Table X. In Exp. 1, about 30% of rats in groups pretreated with MNU showed some degree of mild hyperplasia of the bladder epithelium. A smaller number of rats in these groups

FIG. 2.—Mild diffuse hyperplasia of bladder epithelium from an MNU-pretreated rat given OTS-free saccharin for 50 weeks (Group B). H & E, × 160.

FIG. 3.—Irregular hyperplasia [$P_1$ carcinoma-RMH; JC] of bladder epithelium from an MNU-pretreated rat given OTS-contaminated saccharin for 4 weeks (Group D; interim kill). H & E, × 160.

FIG. 4.—Irregular hyperplasia [$P_1$ carcinoma-RMH; JC] of bladder epithelium from an MNU-pretreated rat given OTS-free saccharin for 40 weeks (Group B). Squamous metaplasia can be seen in one area. H & E, × 160.

FIG. 5.—Papillary hyperplasia [$P_1$ carcinoma-RMH; JC] of bladder epithelium from an MNU-pretreated rat given OTS for 12 weeks (Group E). Mitotic figure arrowed. H & E, × 160.

(a)

(b)

FIG. 6.—(a) Papilloma [papillary carcinoma *in situ*-RMH; JC] of bladder epithelium from an MNU pretreated rat given OTS-free saccharin for 50 weeks (Group B). H & E, × 63. (b) Higher magnification of 6 (a) to show arrangement of epithelial cells. H & E, × 160.

FIG. 7.—Transitional-cell carcinoma of the bladder from an MNU-pretreated rat given OTS-contaminated saccharin for 63 weeks (Group D). H & E, × 160.

FIG. 8.—Transitional-cell carcinoma of the bladder with invasion of underlying muscle, from an MNU-pretreated rat given OTS for 99 weeks. L = bladder lumen; E = hyperplastic epithelium; C = transitional-cell carcinoma; ▶ = nests of carcinoma cells in lamina propria and muscle. H & E, × 63.

TABLE IX.—*Interim bladder pathology*

| Finding | Experimental group A | B | C | D | E | F | G |
|---|---|---|---|---|---|---|---|
| | No. of rats examined at each time: | | | | | | |
| | 4 | 4 | 4 | 4 | 4 | 4 | 4 |
| **4 weeks** | | | | | | | |
| Mild focal hyp. | 1 | 1 | 1 | . | . | . | . |
| Mild diffuse hyp. | . | 1 | 1 | 1 | 1 | . | . |
| Irregular hyp.* | 2 | . | 1 | 1 | . | . | . |
| Papillary hyp.* | . | . | . | . | . | . | . |
| **6 months** | | | | | | | |
| Mild focal hyp. | 2 | . | 2 | . | 2 | . | . |
| Mild diffuse hyp. | . | . | . | 1 | . | . | . |
| Irregular hyp.* | . | 2 | . | 1 | . | . | . |
| Papillary hyp.* | . | 1 | . | 1 | . | . | . |
| **12 months** | | | | | | | |
| Mild focal hyp. | 1 | 1 | 1 | . | 2 | . | . |
| Mild diffuse hyp. | 1 | 1 | . | . | . | . | . |
| Irregular hyp.* | . | . | . | . | . | . | . |
| Papillary hyp.* | . | . | . | . | . | . | . |
| Proliferation of spindle cells in lamina propria | 1 | . | . | . | . | . | . |
| Transitional-cell carcinoma | . | . | . | 1 | . | . | . |

* 9/9 lesions classified as $P_1$ carcinomas by 2 authors.
hyp = hyperplasia.

developed more severe proliferative lesions, the incidence of these lesions being significantly higher in groups given saccharin (B and D). Although 2 authors classified these proliferative changes as irregular or papillary hyperplasias, the other 2 authors diagnosed 15/19 cases as carcinomas, Stages PIS, P1a, or P1b (WHO classification, 1973).

Unequivocal bladder carcinomas were found in all groups pretreated with MNU (Fig. 7) and a small number of connective-tissue neoplasms occurred in the same groups. Invasion of the bladder musculature was seen in only 6 instances (Fig. 8) and metastases to other organs were never seen. The incidence of neoplasia was not significantly different in any of the groups pretreated with MNU, irrespective of the type of classification used. However, neoplasms were first detected in the saccharin-treated groups (B and D) and the mean latent period was also shorter in these groups (Table XI).

TABLE X.—*Terminal bladder pathology*

| Finding | Exp. 1 A | B | C | D | E | F | G | Exp. 2 H | I | J |
|---|---|---|---|---|---|---|---|---|---|---|
| | No. of rats examined: | | | | | | | No. of rats examined | | |
| | 48 | 49 | 48 | 44 | 47 | 50 | 48 | 49 | 49 | 50 |
| Macroscopic calculus | 1 | . | 2 | 2 | . | . | . | . | 8 | . |
| Necrosis and epithelial calcification | 1 | 1 | 4 | 1 | 5 | . | . | . | . | . |
| Necrosis and inflammatory cell infiltration | 1 | . | 1 | . | . | . | . | . | 1 | 1 |
| Foci of lymphocytes in lamina propria | . | . | 1 | . | . | . | . | . | 1 | . |
| Spindle-cell proliferation in lamina propria | . | 1 | 2 | 2 | . | . | . | . | 2 | . |
| Hyperplasia: | | | | | | | | | | |
| Mild focal | 11 | 14 | 5 | 5 | 10 | . | . | 1 | 10 | 1 |
| Mild diffuse | 4 | 4 | 8 | 12 | 5 | . | . | . | 1 | . |
| Irregular* | 2 | 5 | 1 | 5 | . | . | . | . | 2 | . |
| Papillary* | . | . | 1 | 3 | 2 | . | . | . | 3 | . |
| Neoplasia: | | | | | | | | | | |
| Transitional-cell papilloma† | 5 | 7 | 4 | 5 | 2 | . | . | . | 1 | . |
| Transitional-cell ca. | 6 | 5 | 7 | 3 | 7 | . | . | 0 | 14 | . |
| Transitional-cell ca. with muscle invasion | . | . | 1 | 2 | . | . | . | . | 3 | . |
| Squamous-cell ca. with muscle invasion | 1 | . | 1 | 1 | . | . | . | . | . | . |
| Leiomyosarcoma | 2 | 1 | . | . | . | . | . | . | . | . |
| Mesothelioma | . | 1 | . | . | . | . | . | . | . | . |
| Fibroma | . | . | . | 2 | . | . | . | . | . | . |
| Angioma | . | . | . | . | 1 | . | . | . | . | . |
| Angiosarcoma | . | . | . | . | . | . | . | . | 2 | . |

* 15/19 (Exp. 1) and 5/5 (Exp. 2) lesions classified as $P_1$ carcinomas by 2 authors.
† Classified as papillary carcinoma *in situ* by 2 authors.
The figures represent absolute numbers.

TABLE XI.—*Summary of proliferative lesions in the bladder*

| Group | No. of rats examined | Mild epithelial hyperplasia (%) | Marked epithelial hyperplasia (%) | Epithelial neoplasms (%) [carcinomas −RMH; JC] | Connective tissue neoplasms (%) | Rats with neoplasms (%) | Mean latent period (Week) |
|---|---|---|---|---|---|---|---|
| Exp. 1 | | | | | | | |
| A | 48 | 31 | 4 | 25 [29] | 4 | 27 [31] | 87 |
| B | 49 | 37 | 10 | 25 [33] | 4 | 29 [37] | 55 |
| C | 48 | 27 | 4 | 27 [31] | 0 | 27 [31] | 76 |
| D | 44 | 39 | 18* | 25 [36] | 5 | 25 [36] | 52 |
| E | 47 | 32 | 4 | 19 [23] | 2 | 21 [26] | 95 |
| F | 50 | . | . | . | . | . | . |
| G | 48 | . | . | . | . | . | . |
| Exp. 2 | | | | | | | |
| H | 49 | 2 | . | . | . | . | . |
| I | 49 | 23 | 9 | 37 [43] | 4 | 38 [45] | 71 |
| J | 50 | 2 | . | . | . | . | . |

* Differ significantly from corresponding control (Fisher's Exact Test $P < 0{\cdot}05$).

Bladder calculi were infrequent or absent in all groups pretreated with MNU. Untreated controls (F) and rats given OTS alone (G) showed no bladder pathology.

In Exp. 2, pretreatment with MNU (Group I) elicited similar pathological changes to those already described, but more animals were affected (Table X). Treatment with saccharin or OTS alone (Group H and J respectively) elicited mild hyperplasia in 2% of rats, but marked hyperplastic changes, neoplasia and calculi were absent.

The proliferative changes in the bladder obtained in both experiments are summarized in Table XI.

### DISCUSSION

Previous work has shown that prolonged administration of saccharin, in the diet or drinking water, to rats pretreated with a non-carcinogenic dose of MNU (Hicks *et al.*, 1973*a*, 1975) or a threshold dose of FANFT (Cohen *et al.*, 1979) produced a high incidence of tumours of the urinary bladder.

In the present experiments, it had been intended to use a non-carcinogenic dose of MNU also, but in practice the dose administered, although the same as had been used in earlier studies (Hicks *et al.*, 1975) produced bladder neoplasms in 27% of rats in Exp. 1, and 38% in Exp. 2. Hyperplastic lesions of the bladder were found in a further third of the rats in each experiment and pathological changes in the kidney, including a small number of neoplasms, were also found after MNU pretreatment. The problem of the varying carcinogenic potency of different batches of MNU has been discussed elsewhere (Hicks *et al.*, 1978) and similar difficulties have been encountered in studies with FANFT (Jacobs *et al.*, 1977; Cohen *et al.*, 1979). Consequently, in the present experiments the promoting activity of saccharin and OTS was evaluated against a background of pre-existing pathology and, under these conditions, both OTS-contaminated saccharin and OTS-free saccharin failed to increase the number of unequivocal bladder carcinomas. Neoplasms may, however, have occurred earlier in saccharin-treated groups; among the interim kills, the only unequivocal bladder carcinoma was found in a saccharin-treated rat, implying an earlier development of neoplasia; carcinomas were also seen earlier in other rats from these groups, as a consequence of premature mortality. However, mortality may have been associated with the severe kidney pathology present in these animals and therefore it is not possible to say with certainty that the time to tumour development was shortened.

Administration of both saccharins did increase the number of hyperplasias, including severe hyperplasias, of the bladder epithelium, seen after MNU pretreatment, the finding being significant with OTS-contaminated saccharin. Despite differing interpretations by the authors of the classification of certain of these proliferative lesions, the conclusion was unanimous that they occurred more frequently in the saccharin-treated animals than in those receiving MNU alone.

The variable interpretation of certain bladder lesions reflects the absence of a generally agreed system of classification for rat bladder neoplasia, the problem of assessing invasion of the underlying connective tissue, and our incomplete knowledge of the behaviour of such proliferative lesions in the rat bladder. There is no doubt that bladder neoplasia induced by a variety of carcinogens is often preceded by hyperplastic lesions that are papillary or nodular in growth pattern (Tiltman & Friedell, 1971; Kunze *et al.*, 1976) but it is possible that some such lesions are not progressive, or even reversible, and that the reversible and irreversible hyperplasias induced by carcinogens cannot be distinguished histologically. Moreover, similar proliferative lesions have been found in regenerating epithelium not associated with neoplasia (Shirai *et al.*, 1977). However, the marked proliferative lesions seen in the present experiments have been regarded by some (Hicks & Chowaniec, 1978) as irreversible conditions, which in many cases show signs of early stromal invasion (P1a or b carcinomas). If this is correct, then preneoplasia develops very early; these lesions were identified in moribund rats killed at 1 and 6 weeks after MNU treatment and in 4-week interim kills. Further experimental work is required in this area before such problems can be resolved.

The failure of saccharin to increase the number of bladder neoplasms after treatment with a carcinogenic dose of MNU is in keeping with the recent results of Mohr *et al.* (1978) who showed that if the initiating agent was used at a dose that itself produced neoplasia, no promoting effect of saccharin could be demonstrated. These workers observed that daily ingestion of 2% saccharin did not alter the incidence of bladder carcinoma in groups pretreated with a dose of MNU that produced neoplasia in 40% of rats. Our findings, and those of Mohr *et al.* (1978) are analagous to the early work on 2-stage carcinogenesis in mouse skin (Berenblum, 1941). In these experiments, it was found that the tumour incidence after a carcinogenic dose of benzpyrene could not be increased by subsequent application of croton oil, and Berenblum concluded that, in order to demonstrate 2-stage carcinogenesis, the initiating agent must be used at a threshold, or subcarcinogenic dose.

The current experiments were designed to elucidate the role of the saccharin contaminant OTS in the production of bladder cancer. Apart from the consequences arising from a reduced water intake (*i.e.* reduced body weight, food intake and concentrated urine) addition of OTS to the drinking water did not alter the incidence of toxicological or pathological changes induced by MNU alone; the higher incidence of bladder neoplasia observed in rats pretreated with MNU and given OTS in the diet (Exp. 2) was not significant; a different batch of MNU was used in this experiment, with a greater carcinogenic potential than the batch used in Exp. 1 (Hicks *et al.*, 1978). Administration of OTS alone, in the diet or drinking water did not produce either bladder hyperplasia or neoplasia. These results are in contrast to the findings of Schmähl (1978) who reported that 5/76 rats fed 200 mg/kg/

day OTS and 3/75 rats given 20 mg/kg/day, developed bladder neoplasia. However, in a 2-generation study by Arnold *et al.* (1977*a*) dietary administration of OTS at levels of 2·5, 25 or 250 mg/kg/day did not induce an increase in bladder neoplasia in either the $F_0$ or the $F_1$ generation.

OTS is a sulphonamide, and has been shown to inhibit carbonic anhydrase *in vitro* (Kinzer, 1973). Similar enzyme inhibition *in vivo* could favour the formation of urinary calculi by increasing urinary pH, and it has been suggested that urolithiasis may have been a factor in the previously reported neoplastic response of the rat bladder to saccharin.

In our experiments, no consistent changes in urinary pH or crystalluria were observed in any of the treatment groups, and only a very small number of rats developed bladder calculi. In Exp. 1, 4 groups of rats, as a result of reduced water intakes, produced concentrated urines for most of the experimental period. Although increased crystalluria and calculus formation might have developed under such conditions, in practice they did not. Furthermore, bladders from rats producing concentrated urine Groups E and G) were histologically similar to bladders from control groups (A and F), which secreted urine of normal specific gravity. Thus the heightened proliferative response in the bladders of rats given saccharin in the drinking water (B and D) could not be attributed to the concentrated urine secreted by rats in these groups. Collectively, these observations lend no support to the hypothesis that increased urinary concentration, crystalluria or urolithiasis play an important role in the development of bladder neoplasia after saccharin administration.

However, in contrast to the situation in the bladder, the increased kidney pelvic epithelial hyperplasia, seen in groups given saccharin alone, or given saccharin after MNU pretreatment, was accompanied by an increased incidence of mineralization in the kidney. Similar findings have been reported previously in rats treated with contaminated saccharin alone (Chowaniec & Hicks, 1979). The results of the current experiments using OTS-free saccharin confirm that the increased kidney hyperplasia and mineralization in saccharin-treated rats with or without MNU administration was associated with saccharin *per se*, and not with the presence of OTS.

The mechanism of saccharin carcinogenicity in the rat bladder is not yet understood. However from the results of the work reported here, there is no evidence that OTS contamination plays a causative role in saccharin-induced pathology. This conclusion supports the findings of Cohen *et al.* (1979) who used OTS-free saccharin to promote bladder carcinogenesis after treatment with a threshold dose of FANFT.

This work was supported by Grant No. G/973/769 from the Medical Research Council.

The authors gratefully acknowledge the invaluable assistance of Mrs Gwyneth Wexler for statistical analyses, Mr John Evans for general pathology, and the staffs of the animal unit, safety evaluation and pathology sections, BIBRA, for expert technical help.

### REFERENCES

Arnold, D. L., Charbonneau, S. M., Moodie, C. A. & Munro, I. C. (1977*a*) Long term toxicity study with orthotoluene sulphonamide and saccharin. *Toxicol. Appl. Pharmacol.*, **41**, 164 (Abst.).

Arnold, D. L., Moodie, C. A., Stavric, B., Stoltz, D. R., Grice, H. C. & Munro, I. C. (1977*b*) Canadian saccharin study. *Science*, **197**, 320.

Berenblum, I. (1941) The mechanisms of carcinogenesis. A study of the significance of cocarcinogenic action and related phenomena. *Cancer Res.*, **1**, 807.

Chowaniec, J. & Hicks, R. M. (1979) Response of the rat to saccharin with particular reference to the urinary bladder. *Br. J. Cancer*, **39**, 355.

Cohen, S. M., Arai, M., Jacobs, J. B. & Friedell, G. H. (1979) Promoting effect of saccharin and DL-tryptophan in urinary bladder carcinogenesis. *Cancer Res.*, **39**, 1207.

Friedman, M. (1937) The use of ranks to avoid the assumption of normality implicit in the analysis of variance. *J. Am. Stat. Assoc.*, **32**, 675.

Hicks, R. M. & Chowaniec, J. (1977) The importance of synergy between weak carcinogens in the induction of bladder cancer in experimental animals and humans. *Cancer Res.*, **37**, 2943.

Hicks, R. M. & Chowaniec, J. (1978) Experimental induction, histology and ultrastructure of hyperplasia and neoplasia of the urinary bladder epithelium. In *International Review of Experimental Pathology*, Ed. Richter, & Epstein. New York: Academic Press. p. 199.

HICKS, R. M., CHOWANIEC, J. & WAKEFIELD, J. ST.J. (1978) The experimental induction of bladder tumours by a two-stage system. In *Mechanisms of Tumour Promotion and Co-carcinogenesis*. Vol. 2. Ed. Slaga. New York: Raven Press. p. 475.

HICKS, R. M., WAKEFIELD, J. ST.J. & CHOWANIEC, J. (1973*a*) Co-carcinogenic action of saccharin in the chemical induction of bladder cancer. *Nature*, **243**, 347.

HICKS, R. M., WAKEFIELD, J. ST.J., & CHOWANIEC, J. (1973*b*) Impurities in saccharin and bladder cancer. *Nature*, **243**, 424.

HICKS, R. M., WAKEFIELD, J. ST.J. & CHOWANIEC, J. (1975) Evaluation of a new model to detect bladder carcinogens or co-carcinogens; Results obtained with saccharin, cyclamate and cyclophosphamide. *Chem-Biol. Interact.*, **11**, 225.

JACOBS, J. B., ARAI, M., COHEN, S. M. & FRIEDELL, G. H. (1977) A long term study of reversible and progressive urinary bladder cancer lesions in rats fed N-[4-(5-nitro-2-furyl)-2-thiazolyl] formamide. *Cancer Res.*, **37**, 2817.

KINZER, G. W. (1973). *Interim Report Scientific Committee of Calorie Control Council.*

KROES, R., PETERS, P. W. J., BERKVENS, J. M., VERSCHUUREN, H. G., DEVIRES, T. & VAN ESCH, G. J. (1977) Long term toxicity and reproduction study (including a teratogenicity study) with cyclamate, saccharin and cyclohexylamine. *Toxicology*, **8**, 285.

KRUSKAL, W. H. & WALLIS, W. A. (1952) Use of ranks in one-criterion variance analysis. *J. Am. Statist. Assoc.*, **47**, 583.

KUNZE, E., SCHAUER, A. & SCHATL, S. (1976) Stages of transformation in the development of N-butyl-N-(4-hydroxybutyl) nitrosamine induced transitional cell carcinomas in the urinary bladder of rats. *Z. Krebsforsch*, **87**, 139.

LESSEL, B. (1971) Carcinogenic and teratogenic aspects of saccharin. Proc. *III Int. Cong. Fd. Sci. Technol.* 1970. Eds Stewart & Willey. Chicago: Inst. Food Technologists. p. 764.

MOHR, U., GREEN, U., ALTHOFF, J. & SCHNEIDER, P. (1978) Syncarcinogenic action of saccharin and sodium cyclamate in the induction of bladder tumours in MNU-pretreated rats. In *Proc. Eur. Res. Gp. Oral Biol. Conf. Health and Sugar Substitutes.* Basel: Karger. p. 64.

MUNRO, I. C., MOODIE, C. A., KREWSKI, D. & GRICE, H. C. (1975) A carcinogenicity study of commercial saccharin in the rat. *Toxicol. Appl. Pharmacol.* **32**, 513.

PETO, R. & PIKE, M. C. (1973) Conversion of the approximation $\Sigma(0\text{-}E)^2/E$ in the logrank test for survival data or tumour incidence data. *Biometrics*, **29**, 579.

SCHMÄHL, D. (1973) Fehlen einer kanzerogenen Wirkung von Cyclamat, Cyclohexylamin, und Saccharin beis Ratten. *Arzneim-Forsch.*, **23**, 1466.

SCHMÄHL, D. (1978) Experiments on the carcinogenic effect of ortho-toluol-sulfonamid (OTS). *Z. Krebsforsch*, **91**, 19.

SHIRAI, T., COHEN, S. M., FUKUSHIMA, S. & ITO, N. (1977) Production of reversible papillary proliferation of the urinary bladder in rats. *Gann*, **68**, 521.

TAYLOR, J. M. & FRIEDMAN, L. (1974) Combined chronic feeding and three generation reproduction study of sodium saccharin in the rat. *Toxicol. Appl. Pharmacol.*, **29**, 154 (Abst).

TILTMAN, A. J. & FRIEDELL, G. H. (1971) The histogenesis of experimental bladder cancer. *Invest. Urol.*, **9**, 218.

TISDEL, M. O., NEES, P. O., HARRIS, D. L. & DERSE, P. H. (1974). Long term feeding of saccharin in rats. In *Symposium Sweetners*, Ed. Inglett. Westpart. p. 145. Further data in *Cancer Testing Technology and Saccharin* (1977) Washington, D.C.: U.S. Govt Printing Office. p. 57.

W.H.O. (1973) International histological classification of tumours: Histological typing of urinary bladder tumours. Ed. Mostofi, Geneva. W.H.O.